# Patchy Micelles with a Crystalline Core: Self-Assembly Concepts, Properties, and Applications

**DOI:** 10.3390/polym13091481

**Published:** 2021-05-04

**Authors:** Christian Hils, Ian Manners, Judith Schöbel, Holger Schmalz

**Affiliations:** 1 Macromolecular Chemistry II, University of Bayreuth, Universitätsstraße 30, 95440 Bayreuth, Germany; christian.hils@uni-bayreuth.de; 2 Department of Chemistry, University of Victoria, 3800 Finnerty Road, Victoria, BC V8P 5C2, Canada; manners@uvic.ca; 3 Fraunhofer Institute for Applied Polymer Research IAP, Geiselbergstraße 69, 14476 Potsdam-Golm, Germany; 4 Bavarian Polymer Institute (BPI), University of Bayreuth, Universitätsstraße 30, 95440 Bayreuth, Germany

**Keywords:** crystallization-driven self-assembly (CDSA), crystalline-core micelles, patchy micelles, block copolymers

## Abstract

Crystallization-driven self-assembly (CDSA) of block copolymers bearing one crystallizable block has emerged to be a powerful and highly relevant method for the production of one- and two-dimensional micellar assemblies with controlled length, shape, and corona chemistries. This gives access to a multitude of potential applications, from hierarchical self-assembly to complex superstructures, catalysis, sensing, nanomedicine, nanoelectronics, and surface functionalization. Related to these applications, patchy crystalline-core micelles, with their unique, nanometer-sized, alternating corona segmentation, are highly interesting, as this feature provides striking advantages concerning interfacial activity, functionalization, and confinement effects. Hence, this review aims to provide an overview of the current state of the art with respect to self-assembly concepts, properties, and applications of patchy micelles with crystalline cores formed by CDSA. We have also included a more general discussion on the CDSA process and highlight block-type co-micelles as a special type of patchy micelle, due to similarities of the corona structure if the size of the blocks is well below 100 nm.

## 1. Introduction

**Figure 1:**
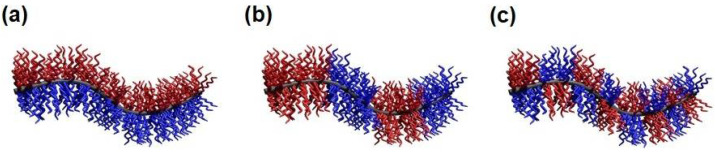
Schematic depiction of a cylindrical (**a**) Janus micelle, (**b**) block co-micelle, and (**c**) patchy micelle.

The solution self-assembly of block copolymers (BCPs) has paved the way to a vast number of micellar assemblies of various shapes (e.g. spheres, cylinders, vesicles, platelets, core-shell, core-shell-corona, and compartmentalized (core or corona) structures) and hierarchical superstructures, as well as hybrids with fascinating applications in drug delivery and release, as emulsifiers/blend compatibilizers, in nanoelectronics, as responsive materials (temperature, pH, light), templates for nanoparticles, in heterogeneous catalysis, etc. [1,2,3,4,5,6]. A key prerequisite for controlling/programming the solution self-assembly is the synthesis of well-defined diblock and triblock (linear, star-shaped, ABA- or ABC-type) copolymers via controlled or living polymerization techniques, such as living anionic polymerization, reversible addition−fragmentation chain transfer, nitroxide-mediated, and atom transfer radical polymerization [5,6,7,8,9]. In general, anisotropic polymer micelles can be divided into three main categories: multicompartment core micelles (MCMs), surface-compartmentalized micelles, and a combination of both [2]. MCMs are generally defined as micellar assemblies with a solvophilic corona and a microphase-separated solvophobic core. According to the suggestion of Laschewsky et al., a key feature of multicompartment micelles is that the various sub-domains in the micellar core feature substantially different properties to behave as separate compartments [10,11]. MCMs are commonly prepared via hierarchical self-assembly of suitable building blocks, which provide “sticky patches” [12,13,14,15]. Depending on the number and geometrical arrangement (linear, triangular, tetrahedral, etc.) of the “sticky patches”, as well as the volume fraction of the solvophilic block, various spherical, cylindrical, sheet-like, and vesicular MCMs are accessible [16,17,18,19,20,21,22,23,24,25]. For a deeper insight into this highly relevant topic, the reader is referred to recent extensive reviews on MCMs [26,27,28,29,30,31]. Surface-compartmentalized micelles are subdivided into micelles with a Janus-type (two opposing faces with different chemistry or polarity) or patch-like, microphase-separated corona, featuring several compartments of different chemistry or polarity (denoted as patchy micelles), as illustrated in Figure 1 for cylindrical micelles. Here, block co-micelles with a block-like arrangement of several (>2) surface compartments along the cylindrical long axis can be regarded as a special case of patchy micelles. It is noted that AB-type diblock co-micelles also represent Janus-type micelles, where the two opposing faces are arranged perpendicular to the cylindrical long axis. The broken symmetry of Janus particles offers efficient and distinctive means of targeting complex materials by hierarchical self-assembly and realize unique properties and applications, like particulate surfactants, optical nanoprobes, biosensors, self-propulsion, and many more [32,33,34,35,36,37,38,39,40,41].

For the preparation of patchy micelles and polymersomes from amorphous BCPs, three main strategies can be applied: (i) self-assembly of ABC triblock terpolymers in selective solvents for the incompatible A and C blocks [42,43,44,45,46,47,48]; (ii) co-assembly of AB and CD diblock copolymers with selective interactions between the B and C blocks (e.g. hydrogen bonding, ionic interactions, solvophobic interactions) [49,50,51,52], resulting in patchy micelles with an insoluble mixed B/C core; and (iii) co-assembly of AB and BC diblock copolymers [53,54,55,56] where the B block forms the insoluble core. However, mostly spherical micelles or polymersomes with a patchy corona have been reported and only a few reports describe the preparation of one-dimensional (worm-like, cylindrical) assemblies with a patch-like compartmentalized corona, even though theoretical work on mixed polymer brushes predict their existence [57,58,59,60,61]. One of the rare but highly intriguing examples are P*t*BA–*b*–PCEMA–*b*–PGMA (poly(*tert*-butyl acrylate)–*block*–poly(2-cinnamoyloxyethyl methacrylate)–*block*–poly(glyceryl monomethacrylate)) and P*n*BA–*b*–PCEMA–*b*–P*t*BA (P*n*BA: poly(*n*-butyl acrylate)) triblock terpolymers [42,43,45]. For self-assembly, the triblock terpolymers were first dissolved in a good solvent for all blocks (CH_2_Cl_2_, CHCl_3_, or THF), followed by the addition of methanol (non-solvent for the middle block) to induce micelle formation. As an intermediate, cylindrical micelles with a patchy corona were formed first, with the P*t*BA blocks forming small circular patches in a corona mainly consisting of PGMA or P*n*BA. Upon further decreasing the solvent quality for the P*t*BA block (addition of MeOH), these cylinders can form double and triple helices via hierarchical self-assembly. This concept has also been applied to triblock terpolymers with a poly(2-hydroxyethyl methacrylate) middle block, having the potential for further modification by esterification of the pendant hydroxy functions [42]. Besides, crystallization-driven self-assembly (CDSA) is a highly versatile tool for the preparation of well-defined cylindrical micelles of controlled length and length distribution, and has proven as a valuable method for the preparation of patchy cylindrical micelles. 

This review will focus on the recent developments concerning self-assembly strategies for the production of crystalline-core micelles (CCMs) bearing a patchy corona, and will also address their unique properties and potential applications. As stated above, block co-micelles represent a special case of patchy micelles and thus, will be discussed only briefly. This is not only due to the usually larger size and sequential arrangement of surface compartments in the corona, in contrast to the more alternating arrangement in patchy cylindrical micelles (Figure 1b,c), but is also attributed to the substantially different self-assembly procedure. Block co-micelles are commonly prepared by sequential living CDSA of different diblock copolymers, whereas patchy micelles are formed by simultaneous CDSA of diblock copolymer mixtures or CDSA of ABC triblock terpolymers with crystallizable middle blocks. Hence, this review will be divided into four main sections, starting with a general consideration of CDSA. The second part gives a compact overview over self-assembly strategies used to form cylindrical and platelet-like block co-micelles. The different self-assembly concepts for patchy micelles with crystalline cores will be reviewed in the third section, followed by a discussion on properties and applications of these interesting compartmentalized nanostructures.

## 2. Crystallization-Driven Self-Assembly (CDSA)

**Figure 2:**
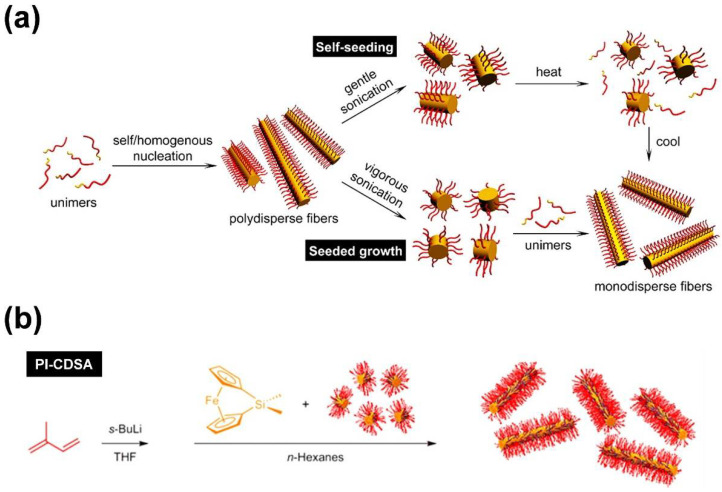
(**a**) Concepts for living CDSA, enabling the production of cylindrical micelles with defined length and narrow length distribution. Self-seeding employing seeds produced by thermal treatment of micelle fragments (top) and seeded growth using small micellar fragments (“stub”-like micelles) as seeds (bottom). (**b**) Living polymerization-induced CDSA (PI-CDSA) utilizing micellar seeds during anionic polymerization of the PFS block. After complete conversion, the reaction was quenched with 4-*tert*-butylphenol. (**a**) Reproduced from [76] with permission of the American Chemical Society (ACS).

As pointed out in the introduction, the preparation of one-dimensional (1D) cylindrical (or worm-like) micelles with controlled dimensions, low-length dispersities, and tailored corona structures and functionalities still remains a challenge in the self-assembly of fully amorphous BCPs. Besides, the introduction of a crystallizable block, which adds an additional and strong driving force for micelle formation, has turned out to be a highly efficient route to solve these issues. Consequently, the self-assembly of such BCPs, bearing crystallizable blocks, is termed crystallization-driven self-assembly (CDSA) [1,62,63]. This field was pioneered by studies on poly(ferrocenyl dimethylsilane) (PFS)-containing BCPs and is gaining increasing importance for the preparation of well-defined 1D and two-dimensional (2D) assemblies, especially since the discovery of living CDSA (Figure 2) [63,64,65,66,67]. Analogous to the living polymerization of monomers, CDSA can proceed in a living manner, employing small micellar fragments as seeds (Figure 2a: seeded growth) for the addition of unimers (molecularly dissolved BCPs with a crystallizable block). In this approach, the micellar seeds, also termed “stub-like” micelles, are produced by vigorous sonication of long, polydisperse cylindrical micelles prepared by conventional CDSA. Owing to its living nature, the length of the produced cylindrical micelles shows a linear dependence on the unimer/seed ratio employed, and length dispersities are very low (*L*_w_/*L*_n_ typically well below 1.1; where *L*_n_ is the number average and *L*_w_ the weight average micelle length). 

Living CDSA can also be realized by using spherical CCMs as seeds [68], by self-seeding [69,70,71] (Figure 2a), and even directly by polymerization-induced CDSA (Figure 2b) [72,73,74], i.e., via polymerization in the presence of seed micelles. The self-seeding approach also uses small micellar fragments that are heated in dispersion to a specific annealing temperature (*T*_a_), where most of the crystalline core is molten/dissolved and only a very minor fraction of crystallites survive. These act as seeds in the subsequent CDSA upon cooling (Figure 2a: self-seeding), and the length of the micelles can be controlled by a proper choice of *T*_a_. If *T*_a_ is too low, the crystalline cores will not melt/dissolve, and the length distribution of the employed micellar fragments remains unchanged. On the other hand, if *T*_a_ is too high, the crystalline cores will melt/dissolve completely, and no crystallites will survive that could act as seeds. As a result, in between these two limiting cases, an increase in micelle length with increasing *T*_a_ is observed, as the fraction of surviving crystallites (seeds) decreases with *T*_a_. This range of self-seeding temperatures can be very restricted, making length control difficult. Another drawback of these seed-based protocols is the low amount of cylindrical micelles that can be produced, as commonly rather dilute solutions have to be used. This can be overcome by the living polymerization-induced CDSA approach, enabling the production of uniform cylindrical micelles with concentrations up to ca. 10–20% (*w*/*w* solids) within a few hours. In a recent report, it was shown that living CDSA can even be stimulated by light, utilizing the photo-induced cis-trans isomerization in oligo(*p*-phenylenevinylene) (OPV)-based BCPs [75].

Living CDSA has paved the way to a myriad of 1D and 2D micellar assemblies of controlled dimensions, including patchy and block co-micelles (both will be addressed in the next sections) [65,68,77,78,79,80], branched micelles [76], platelet-like micelles and co-micelles [81,82,83,84,85,86], and hierarchical assemblies [81,87,88,89,90,91]. Next to BCPs with a PFS block, a variety of other crystallizable polymer blocks were employed in CDSA, e.g. polyethylene (PE) [68,92,93,94], poly(ethylene oxide) [95], polyesters (poly(*ε*-caprolactone) (PCL) or poly(*L*-lactide) (PLLA)) [86,96,97,98,99,100,101], polycarbonate [102], poly(2-*iso*-propyl-2-oxazoline) (P*i*PrOx) [103,104], liquid crystalline polymers [71,105], poly(vinylidene fluoride) [106], polypeptoids [107,108], and various conjugated polymers (e.g., poly(3-hexyl thiophene) (P3HT) and OPV) [75,109,110,111,112,113].

## 3. Short Excursion on Block Co-Micelles

**Figure 3:**
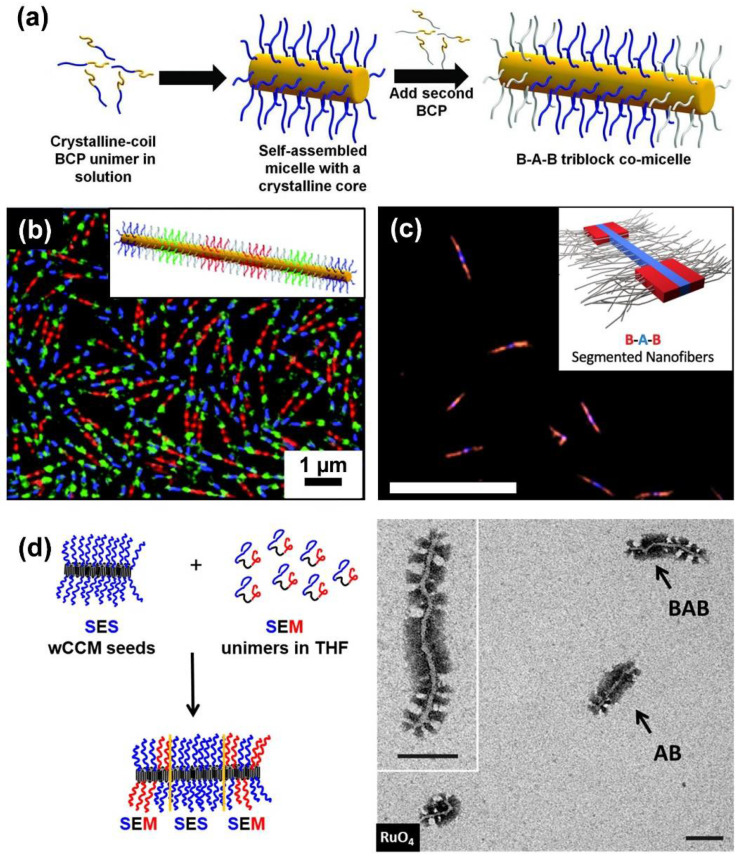
(**a**) Formation of B–A–B triblock co-micelles via sequential living CDSA in selective solvents for the corona blocks. (**b**) Structured illumination microscopy image of symmetrical 11-block co-micelles with red, green, and blue fluorescent corona blocks separated by non-fluorescent PDMS spacer blocks. (**c**) Laser-scanning confocal microscopy image of solid-state, donor–acceptor, coaxial heterojunction nanowires based on B–A–B segmented nanofibers with a semi-crystalline PDHF core (depicted in blue) and a semi-crystalline P3EHT shell (depicted in red) in the outer corona blocks, taken with both blue (PDHF) and red (P3EHT) channels (scale bar: 10 µm). Blue emission from the central PDHF core, as well as red/orange emission from the outer P3EHT segments, due to Förster resonance energy transfer (FRET) were observed. (**d**) Schematic depiction of the formation of B–A–B triblock co-micelles with patchy outer corona blocks, starting from SES wCCMs as seed micelles and subsequent living CDSA of SEM unimers in THF (left) and corresponding TEM image of patchy block co-micelles (scale bar: 100 nm). (**a**) Reproduced from [79] with permission of the American Association for the Advancement of Science (AAAS), (**b**) reproduced from [67] with permission of the Royal Society of Chemistry (RSC), (**c**) reproduced from [105], and (**d**) reproduced from [68] with permission of ACS.

Block co-micelles represent a special type of patchy CCM, because of the sequential arrangement of surface compartments and the precisely adjustable size of the blocks, usually leading to larger corona segments than commonly observed for patchy CCMs. Analogous to the synthesis of BCPs, block co-micelles are produced by sequential living CDSA. The characteristic of this process is that the micelles’ termini remain “active” after unimer addition is completed. Consequently, addition of a different type of unimer leads to the formation of a blocky structure (Figure 3a) [65,114]. This feature allows for precise control over the block length by adjusting the amount of added unimer.

Similar to living polymerization techniques, in which the reactivity of the first monomer limits the choice of a second monomer, unimers need to fulfill certain requirements for successful co-crystallization. For example, the micellar cores need to be compatible for epitaxial crystallization, i.e., they should exhibit a similar crystal lattice spacing [115,116]. A common way to fulfill this prerequisite is the use of diblock copolymers bearing the same crystallizable block that induces homoepitaxial growth, as shown first for PFS-containing diblock copolymers to produce B–A–B triblock co-micelles [65]. Within this work, PFS–*b*–polyisoprene (PFS–*b*–PI) cylindrical micelles served as seeds for the nucleation of PFS–*b*–polymethylvinylsilane (PFS–*b*–PMVS) and PFS–*b*–polydimethylsiloxane (PFS–*b*–PDMS) unimers, respectively. For heteroepitaxial growth, different PFS-containing seed micelles were applied to induce CDSA of polyferrocenylgermane (PFG)-containing diblock copolymers [73,83,89]. The crystal lattice spacing of the two core-forming blocks only differs by about 6%, enabling the formation of tri- and pentablock co-micelles as well as 2D co-assemblies.

Living CDSA has opened the door to a huge variety of one dimensional, PFS-containing block co-micelles with tailored numbers, lengths, and composition of corona blocks [114,117,118,119,120,121]. Centrosymmetric and non-centrosymmetric block co-micelles are accessible, and give rise to broad structural complexity [79]. In particular, the introduction of fluorescent corona blocks marks an important step in the development of block co-micelles, since this enables the formation of barcode and RGB micelles (Figure 3b) [67,77,122]. Up to that point, the fabrication of cylindrical nanomaterials with precise, color-tunable compartments of predictable length and number was challenging. Moreover, it is possible to induce fluorescence in the semicrystalline core-forming block by replacing the PFS block by a poly(di-*n*-hexylfluorene) (PDHF) block [78]. B–A–B triblock co-micelles with PDHF core and P3HT outer corona blocks were found to show long-range exciton transport (>200 nm). Inducing secondary crystallization of a poly(3-(2’-hexylethyl)thiophene) (P3EHT) corona block even rendered solid-state donor–acceptor heterojunctions possible (Figure 3c) [105]. These materials bear a high potential for applications in optoelectronics, device fabrication, and sensing [123].

Several other semicrystalline, core-forming blocks—for example, PFG [73,83,89], polycarbonate [102,124], poly(3-heptylselenophene) [109], P3HT [125], OPV [75,126,127], PLLA [128], and PE [68]—were used for the production of block co-micelles. As an example, sequential living CDSA of a polystyrene–*block*–polyethylene–*block*–polystyrene (PS–*b*–PE–*b*–PS; SES) triblock copolymer with a PS–*b*–PE–*b*–PMMA (SEM; PMMA: poly(methyl methacrylate)) triblock terpolymer yielded B–A–B- or A–B–A-type triblock co-micelles with patchy outer or inner B blocks, respectively (Figure 3d) [68]. Interestingly, the choice of seed micelles was crucial for the successful formation of triblock co-micelles, as worm-like SES micelles are accessible on both micelle ends for epitaxial growth, whereas patchy, worm-like SEM micelles show diverse growth behavior, which is predefined by the arrangement of the corona chains at the micelles’ ends.

**Figure 4:**
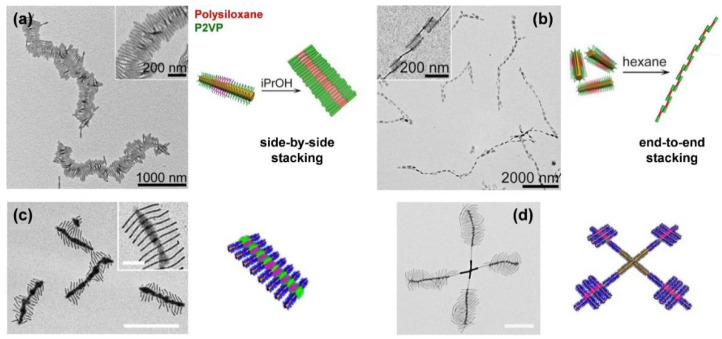
One-dimensional supermicelles by (**a**) side-by-side and (**b**) end-to-end stacking in selective solvents for the outer and middle corona block of B–A–B triblock co-micelles (PFS core), respectively. (**c**) “Shish-kebab” supermicelles (scale bar = 1 µm, inset = 200 nm) formed by hydrogen-bond (H-bond)-mediated co-assembly of an H-bond donor homopolymer (hydroxyl-functionalized poly(vinylmethylsiloxane (PMVSOH), colored in pink) with B–A–B triblock co-micelles with “neutral” outer corona blocks (poly(*t*-butyl acrylate) (P*t*BA), colored in blue, no H-bond interactions) and an H-bond acceptor middle corona block (P2VP, colored in green). (**d**) “Windmill”-like supermicelles via living CDSA of a PFS-*b*-P*t*BA diblock copolymer from “cross” supermicelles (scale bar = 0.5 µm). The “cross” supermicelles featured an H-bond acceptor corona block (P2VP) at the termini, onto which short CCMs with H-bond donor corona blocks (PMVSOH) that served as seeds for the subsequent living CDSA of the PFS-*b*-P*t*BA diblock copolymer (color code identical to (**c**)) were immobilized. (**a**,**b**) reproduced from [88] with permission of AAAS, (**c**) reproduced from [133] with permission of ACS, and (**d**) from [132] with permission of Springer Nature.

The scope of complex micellar assemblies is further extended by hierarchical self-assembly, using block co-micelles as building blocks for the formation of 2D and three-dimensional (3D) superstructures. There are different strategies to realize hierarchical assemblies—for example, coordination-driven co-assembly [129] or dialysis of amphiphilic block co-micelles against selective solvents, enabling highly efficient side-by-side or end-to-end stacking (Figure 4a,b) [88,130,131], or spatially confined hydrogen-bonding interactions [132,133]. The latter opens access to numerous hierarchical 2D morphologies, such as “I”-shaped, cross, shish-kebab (Figure 4c) or windmill-like (Figure 4d) structures, by precisely tailored interactions between hydrogen donor and hydrogen acceptor units within the block co-micelles. However, not only the attractive interactions by hydrogen-bonding have to be taken into account, but also repulsive interactions caused by steric hindrance of the corona chains. To overcome this problem, tuning the length of the hydrogen acceptor blocks has proven to be a suitable solution, rendering 3D assemblies possible. It is noted that 2D platelet-like hierarchical superstructures, as well as more complex micelle architectures like double- and single-headed, spear-like micelles [90], scarf-like micelles [89], diamond-fiber hybrid structures [81], or platelets with various shapes (rectangular, quasi-hexagonal, and diamond platelet micelles) [82,83,84,85] are accessible.

## 4. Self-Assembly Concepts for Patchy Micelles with Crystalline Cores

### 4.1. CDSA of Linear and Star-Shaped Triblock Terpolymers

The most widely used route toward crystalline-core patchy micelles is the CDSA of linear ABC triblock terpolymers with a crystallizable middle block (Table 1) [134]. In contrast to block co-micelles, where the sequential living CDSA of different diblock copolymers results in a block-type segmentation of the corona, the incompatibility of the corona-forming blocks is the driving force for corona segregation in CDSA of triblock terpolymers. This affects the average width of the patches and leads to an alternating, chess-board-like arrangement of the corona patches [135]. Worm-like CCMs (wCCMs) with a patchy corona were first reported in 2008 for triblock terpolymers with a semicrystalline PE middle block and two amorphous outer blocks, namely PS and PMMA (SEM) [93]. Since patchy, worm-like (or cylindrical) CCMs based on these triblock terpolymers have been intensively studied, the self-assembly mechanism will be elucidated in detail on this example.

**Figure 5:**
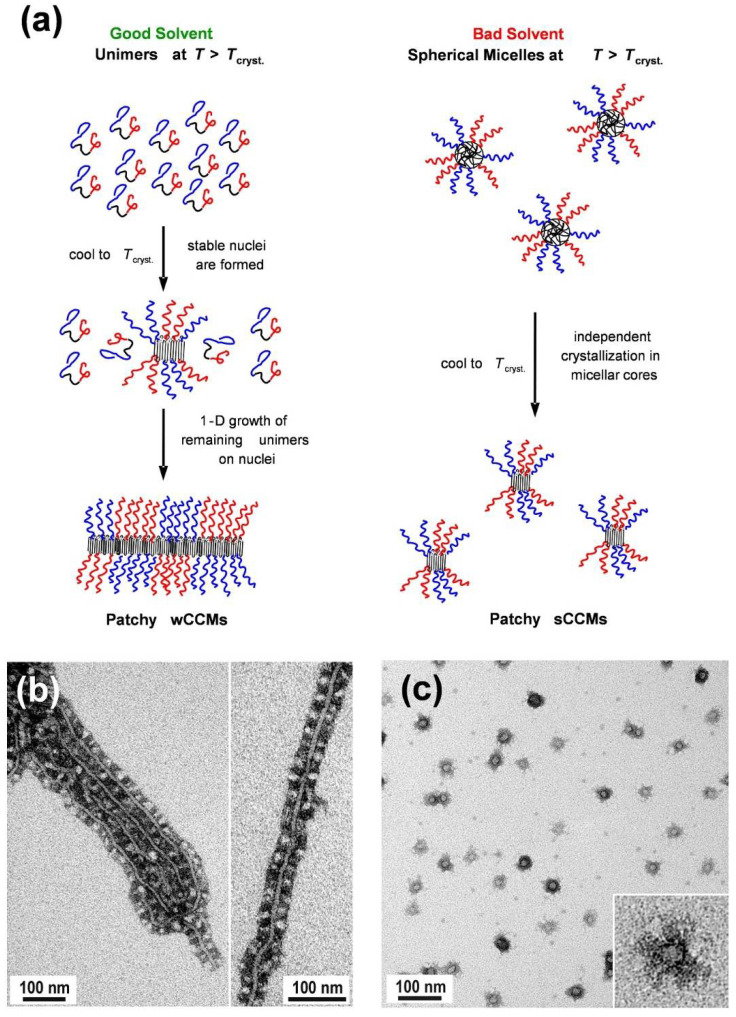
(**a**) Schematic representation of the proposed mechanism for the formation of patchy worm-like and spherical crystalline-core micelles (wCCMs and sCCMs, respectively) from SEM triblock terpolymers (PS blocks are represented in blue, PE in black, and PMMA in red). TEM images of (**b**) patchy S_340_E_700_M_360_ wCCMs prepared by CDSA in THF and subsequent annealing at 45 °C for 3 h, and (**c**) patchy S_340_E_700_M_360_ sCCMs formed in dimethylacetamide (subscripts denote the respective average degrees of polymerization, PS was selectively stained with RuO_4_ vapor and appears dark). Reproduced from [94] with permission of ACS.

Initially, the SEM triblock terpolymers are placed in a good solvent for the amorphous blocks and heated above the melting temperature of the semicrystalline PE block in the given solvent (Figure 5a) [94]. Depending on the solvent quality for the PE middle block, different micelle morphologies are formed. In good solvents for the molten PE block (for example, THF or toluene), the triblock terpolymers are molecularly dissolved, i.e., unimers are formed. In bad solvents for PE (for example 1,4-dioxane), the molten PE block collapses, and spherical micelles with an amorphous (molten) PE core are observed. Cooling of the corresponding unimer solution (in good solvents) or dispersion of spherical micelles (bad solvents) results in the nucleation of PE crystallization. In good solvents, the nuclei are stable and able to initiate the bidirectional, 1D epitaxial growth of the remaining unimers to generate wCCMs. However, in bad solvents, the spherical shape of the micelles dictates the final morphology of the CCMs. Consequently, confined crystallization of PE in the respective micellar cores leads to the generation of spherical CCMs. In both cases, the micelle corona exhibits a patch-like, microphase-separated (patchy) structure, whereas for wCCMs the patchy structure of the corona is more pronounced (Figure 5b,c). For wCCMs, an almost alternating arrangement of the PS and PMMA patches in the corona can be deduced from transmission electron microscopy (TEM) [94], and was also confirmed by small-angle neutron scattering studies [135].

**Figure 6:**
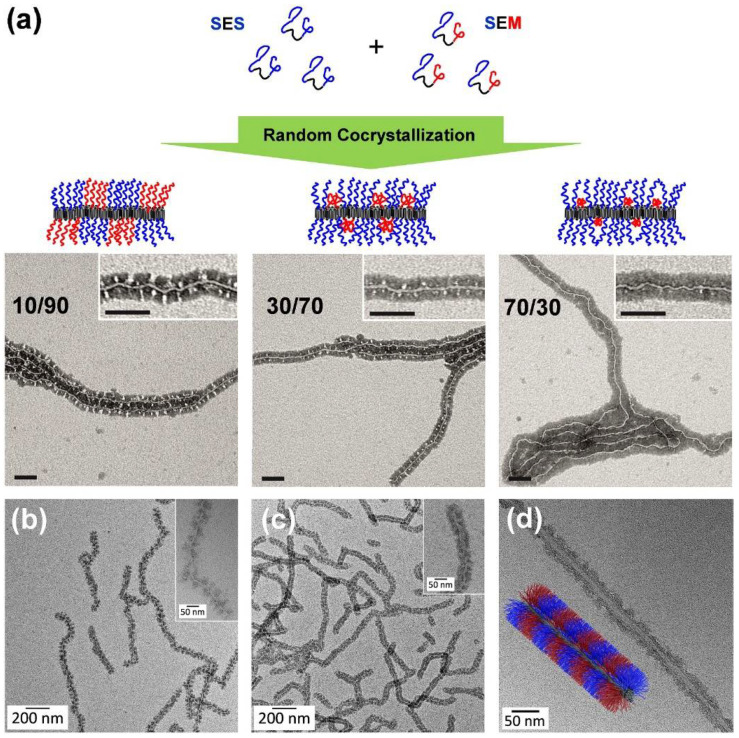
(**a**) Random co-crystallization of a SES triblock copolymer and a SEM triblock terpolymer, in order to tune the size of the corona patches. TEM images of patchy wCCMs obtained by co-crystallization of S_380_E_880_S_390_ with S_340_E_700_M_360_ in THF (subscripts denote the respective average degrees of polymerization), revealing a decreasing size of the bright-appearing PMMA corona patches with an increasing amount of S_380_E_880_S_390_ (scale bars: 100 nm). TEM images of patchy (**b**) S_415_E_830_DMA_420_ and (**c**) S_660_E_1350_DMA_350_ wCCMs (DMA: *N,N*-dimethylaminoethyl methacrylamide), as well as (**d**) 1D patchy hybrids with a CNT core and a patchy PS/PMMA corona prepared by ultrasound-assisted, non-covalent grafting of an SEM triblock terpolymer onto CNTs. For all samples, PS was selectively stained with RuO_4_ vapor and appears dark. (**a**) Reproduced from [136] with permission of Elsevier, (**b**,**c**) reprinted from [138] with permission of RSC, and (**d**) reproduced from [142] with permission of ACS.

A facile way to tailor the sizes of the PS and PMMA corona patches is random co-crystallization of an SEM triblock terpolymer with a corresponding SES triblock copolymer, bearing two PS end blocks [136]. A systematic increase of the SES fraction led to a decrease of the PMMA patch size (Figure 6a). Thus, this approach allows to tune the corona structure by a simple co-assembly without the need to synthesize new triblock terpolymers for each desired corona composition. Another efficient way to modify the corona patches is the introduction of functional groups via selective amidation of the PMMA block in SEM triblock terpolymers with different *N,N*-dialkylethylenediamines [137,138]. CDSA in THF led to patchy wCCMs, for which the patch size and shape could be tuned by varying the block length ratio of the corona blocks (Figure 6b,c) and selective solvent interactions. The functionalized, patchy corona enables an application of these wCCMs as templates for the incorporation of inorganic nanoparticles (NPs), which will be discussed in detail in Section 5.2.

The patchy corona structure of SEM wCCMs can also be transferred to multiwalled carbon nanotubes (CNTs) by a non-covalent grafting approach that forms 1D patchy hybrids (Figure 6d) [142]. In contrast to CDSA, which is commonly used to obtain patchy wCCMs, these patchy hybrids were prepared by an ultrasound-assisted process. Here, the PE block selectively adsorbs onto the CNT surface, while the soluble PS and PMMA blocks form the patchy corona. The driving force for CNT functionalization is the high affinity of the PE block to the CNT surface, which was supported by the use of a SEM triblock terpolymer, which is not able to crystallize at room temperature, but successfully generates patchy CNT hybrids.

**Figure 7:**
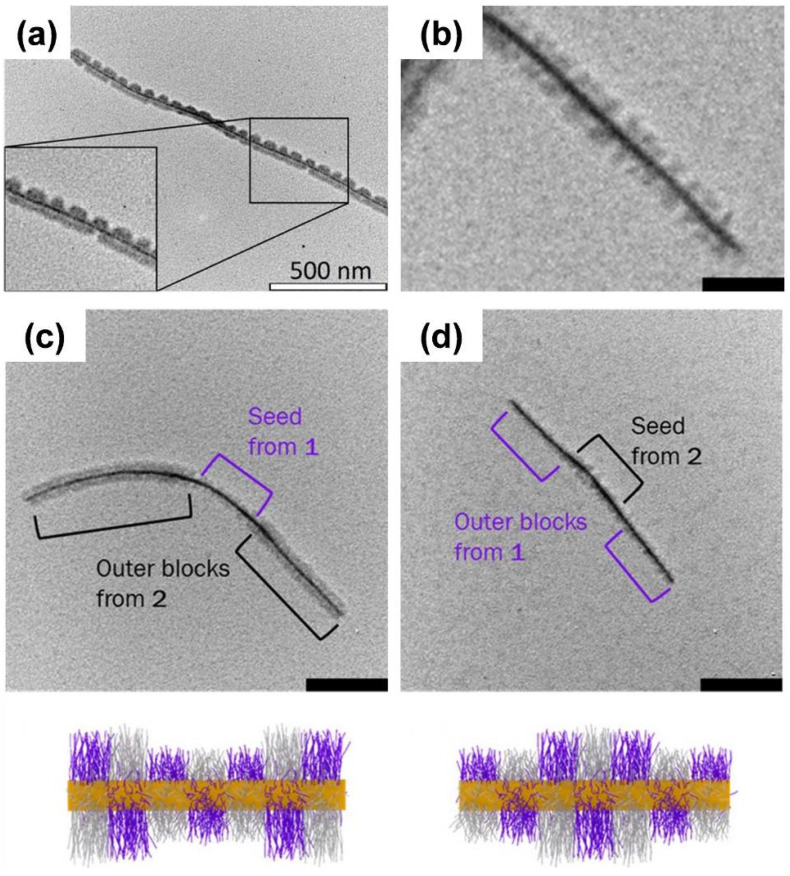
(**a**) Patchy micelles formed by CDSA of a *μ*-SIF (polystyrene–*arm*–polyisoprene–*arm–*poly(ferrocenyl dimethylsilane)) miktoarm star terpolymer in ethyl acetate. (**b**) Patchy cylindrical micelles and (**c**,**d**) patchy ABA-type triblock co-micelles with a crystalline PFS core and a patchy PS/PMMA corona prepared in acetone (scale bars = 100 nm). In (**c**,**d**), triblock terpolymers with PS and PMMA blocks of different lengths were used to alter the width of the patchy corona in the middle and outer blocks of the triblock co-micelles (in the sketches PS is depicted in light grey and PMMA in purple). (**a**) Reprinted from [141], and (**b**–**d**) from [139] with permission of ACS.

Different attempts were made to exchange the PE block with another crystallizable block in order to generate patchy wCCMs. Successful examples are triblock terpolymers of PS–*b*–PFS–*b*–PMMA, PS–*b*–PFS–*b*–PMVS, and PI–*b*–PFS–*b*–PMMA [139,140], as well as *µ*-ABC miktoarm star terpolymers with a crystallizable PFS block (Figure 7a) [141]. The PFS-containing triblock terpolymers were able to undergo a seeded growth protocol for living CDSA in different solvents to form patchy wCCMs of predictable length (Figure 7b). Remarkably, the living CDSA of all triblock terpolymers proceeded rather slowly compared to PFS-containing diblock copolymers, which was attributed to two effects: (i) the comparably high steric hindrance caused by the two corona blocks surrounding the core-forming block, and (ii) the choice of solvent, which did not sufficiently support the crystallization of PFS. For the PS–*b*–PFS–*b*–PMMA triblock terpolymers, the corona chain length (core to total corona block ratio) was varied, and co-crystallization of the resulting triblock terpolymers resulted in block co-micelles with a patchy corona. Interestingly, the different micelle blocks were still discernible by TEM analysis because of the different corona thicknesses (Figure 7c).

### 4.2. Co-Assembly of Diblock Copolymers

**Figure 8:**
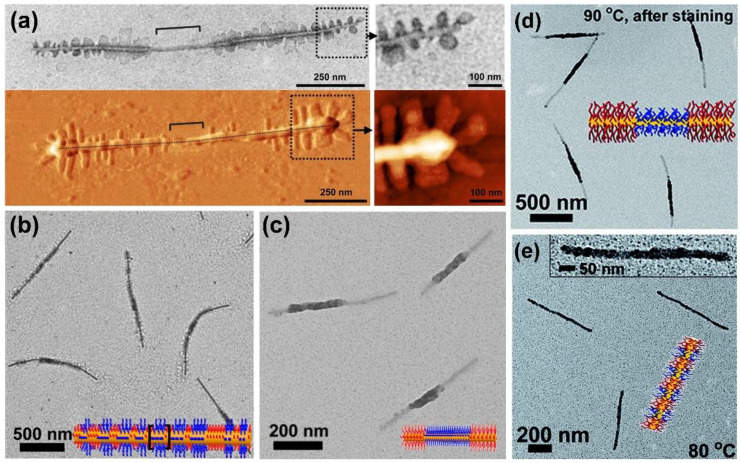
(**a**) TEM (top), as well as AFM topography (bottom left) and phase (bottom right) images of B–A–B triblock co-micelles with patchy end blocks prepared by the co-crystallization of linear and brush-type BCPs with a crystallizable PFS block, employing cylindrical PFS–*b*–PDMS seed micelles. (**b**) Patch-like segmented and (**c**) B–A–B triblock co-micelles produced by controlling the epitaxial growth rate of PFS–*b*–PNiPAM over PFS–*b*–P2VP onto cylindrical PFS–*b*–P2VP seed micelles. Comparable growth rates resulted in patch-like segmentation and dissimilar growth rates in a blocky structure of the corona. (**d**) B–A–B triblock co-micelles and (e) patch-like, segmented co-micelles prepared by synergistic self-seeding of a mixture of short PFS–*b*–PNiPAM and PFS–*b*–P2VP cylindrical micelles. In (**d**), the P2VP middle block corona was selectively stained with platin NPs. (**b**–**e**) In the respective sketches, PFS is colored in light orange, P2VP in blue, and PNiPAM in red. (**a**) Reprinted from [143], (**b**,**c**) reprinted from [145] with permission of ACS, and (**d**,**e**) reproduced from [80] with permission of RSC.

The simultaneous co-assembly of PFS-based diblock copolymers represents an alternative way of producing patchy, cylindrical CCMs, next to the use of synthetically more demanding linear or star-shaped triblock terpolymers (Table 1). However, the corona patches of the resulting micelles are usually arranged in a blocky rather than an alternating manner. Consequently, the micelles produced with this approach represent a special case of patchy CCMs. The first example of these patchy block co-micelles was reported in 2014, and is based on the co-crystallization of linear and brush-type BCPs with a crystallizable PFS block [143]. Starting from a linear PFS–*b*–PMVS diblock copolymer, the PMVS corona block was alkylated via thiol–ene functionalization, in order to yield a brush-type BCP with pendant C18 alkyl chains. The brush-type BCPs showed poor crystallization behavior, due to the steric repulsion of the alkyl moieties. However, simultaneous co-crystallization with the linear BCP, applying cylindrical PFS–*b*–PDMS seed micelles, resulted in a gradual integration of the brush-type unimers. Hence, a patchy corona segmentation of the end blocks was observed for the produced B–A–B triblock co-micelles by TEM and atomic force microscopy (AFM) (Figure 8a).

The preparation of patchy block co-micelles is not limited to sterically demanding co-blocks, but can be induced by a strong difference in the Flory–Huggins interaction parameter between the corona-forming blocks [144]. Blends of PFS–*b*–PDMS with PFS–*b*–PMVS and PFS–*b*–PI, respectively, were co-crystallized, resulting in a blocky corona segmentation. Staining with Karstedt’s catalyst (selective for PI and PMVS) revealed the small corona patches and made two different patch arrangements visible (helical pattern and hemispherical shape). In a subsequent study, the competitive seeded-growth kinetics of the simultaneous co-crystallization of diblock copolymers bearing different corona blocks was investigated [145]. To this end, PFS–*b*–poly(2–vinylpyridine) (PFS–*b*–P2VP) was co-crystallized with two different PFS–*b*–poly(*N*-isopropyl acrylamide) (PFS–*b*–PNiPAM) diblock copolymers using short PFS–*b*–P2VP seed micelles. The length of the PFS block was similar in all used diblock copolymers, but the corona block length of the PFS–*b*–PNiPAM diblock copolymers differed, which affected the epitaxial growth rate of the PFS–*b*–PNiPAM unimers on the seed micelles. If this growth rate was comparable to that of the competing PFS–*b*–P2VP unimers, patchy micelles were observed (Figure 8b). If, on the other hand, the growth rates of the two competing diblock copolymers differed significantly, the formation of block co-micelles was preferred (Figure 8c). Additionally, the epitaxial growth rate of the PFS–*b*–P2VP diblock copolymers was manipulated by quaternization of the P2VP block, which generated a permanent positive charge within the corona chains. Co-crystallization with a PFS–*b*–PNiPAM diblock copolymer, which yielded a patchy structure with the non-quaternized PFS–*b*–P2VP, then led to a blocky arrangement of the patches, which could again be attributed to the differing epitaxial growth rates.

Beyond changes in the epitaxial growth rate by manipulation of the corona chains, the crystallization behavior of the PFS core block can also be altered [80]. A variation in the PFS block length affects the so-called critical dissolution temperature (*T*_c_). This temperature describes the point at which the initial average micelle length doubles upon cooling. Heating a mixture of two different micelle fragments with similar *T*_c_ values to an annealing temperature (*T_a_*), and *T*_a_ < *T*_c_ results in separate micelle fragments. If *T*_a_ is in the range of the *T*_c_ of both micellar fragments, the micellar fragments dissolve partly, and tadpole-shaped fragments are observable. If *T*_a_ > *T*_c_, self-seeding is taking place and the growth kinetics are dictated by the epitaxial growth rates of the two competing unimer types, i.e., a patchy morphology is observed for similar growth rates and a blocky arrangement of the patches results from dissimilar growth rates (Figure 8d). The self-seeding behavior changes if two diblock copolymers with different *T*_c_ values are employed. If *T*_a_ is raised above the *T*_c_ of one of the diblock copolymers, but is still lower than the *T*_c_ of the other diblock copolymer, the diblock copolymer with the lower *T*_c_ will partly or almost fully dissolve and epitaxially grow from the remaining micelle seed fragments of both diblock copolymers. This results in either match stick-like micelles or block co-micelles. If *T*_a_ is increased well above the *T*_c_ of both diblock copolymers, again the growth kinetics determine the final observable corona arrangement—i.e., for similar growth rates, a patchy segmentation is generated (Figure 8e). This concept can also be transferred to mixtures of PFS homopolymers and PFS-based BCPs [146]. Due to the higher *T*_c_ of the PFS homopolymer, a certain fraction of PFS homopolymer crystal fragments will survive upon proper choice of *T*_a_; these fragments then act as seeds upon subsequent cooling and annealing. This not only allows the production of cylindrical micelles of uniform length, but also of well-defined block co-micelles or patchy micelles employing a mixture of PFS with different PFS-based BCPs. An important feature of this approach with respect to applications is the comparably easy scale-up, enabling the production of uniform cylindrical micelles of controlled architecture up to concentrations of 10% (*w*/*w* solids) or more.

## 5. Properties and Applications

### 5.1. Interfacial Activity and Blend Compatibilization

**Figure 9:**
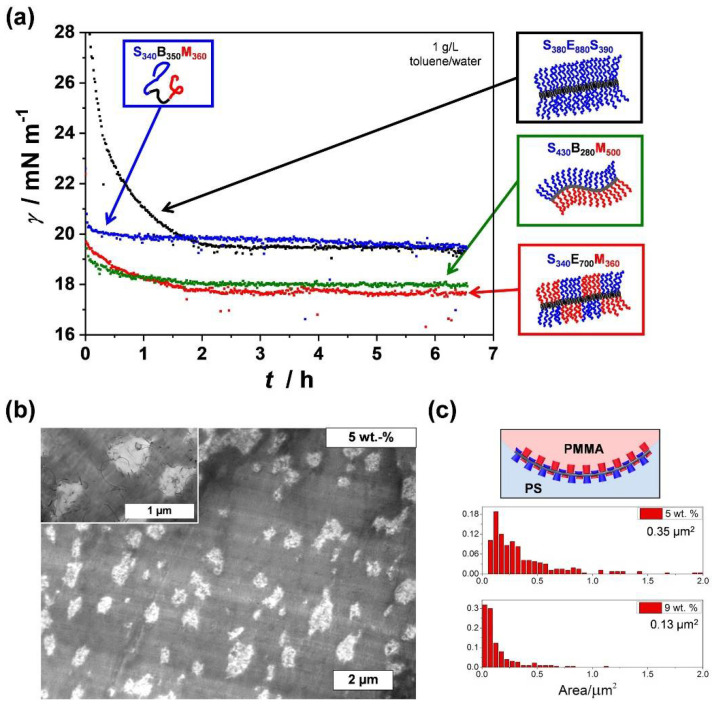
(**a**) Comparison of interfacial tension isotherms of 1 g∙L^−1^ solutions containing SBM unimers, SES wCCMs with a homogeneous PS corona, SEM wCCMs with a patchy PS/PMMA corona, and SBM-based Janus cylinders with opposing PS and PMMA faces (given subscripts correspond to average degrees of polymerization of the respective blocks). (**b**) TEM image of a solvent-cast PS/PMMA blend (80/20 *w*/*w*) compatibilized with 5 wt.%. patchy CNTs (PS/PMMA corona). (**c**) Schematic representation of the adaption of the patchy PS/PMMA corona to the PS/PMMA blend interface by selective collapse/expansion of the incompatible/compatible corona blocks (top) and histograms of PMMA domain areas for blends with 5 wt.%. and 9 wt.%. patchy CNTs (bottom). (**a**) Reproduced from [159] with permission of Elsevier and (**b**,**c**) reproduced from [162] with permission of ACS.

The alternating, patch-like arrangement in the corona of worm-like (or cylindrical) patchy CCMs offers a high potential for a variety of applications. As was shown for amorphous Janus micelles, polymer particles exhibiting two opposing faces made of PS and PMMA (or poly(*tert*-butyl methacrylate)) serve as excellent particulate surfactants and compatibilizers in polymer blends [147,148,149,150,151,152,153,154,155,156,157,158]. This originates from the unique interfacial activity of these materials [38]. Patchy wCCMs were proven to show not only a superior interfacial activity compared to cylindrical micelles with a homogeneous PS corona, but also an identical interfacial activity compared to that of Janus micelles at a water–toluene interface (Figure 9a) [159]. Although Janus particles consist of only two clearly separated compartments (or faces), which facilitates the orientation at interfaces, the unique corona structure of patchy micelles is able to adapt to the requirements of the interface, i.e., the respective insoluble block will collapse and the soluble block will expand. Depending on the molecular weight of the corona-forming blocks and thus, the thickness of the corona, the interfacial activity could be tuned, and was shown to increase with thickness (at constant micelle length), which is in good agreement with theoretical predictions [160]. Interestingly, patchy SEM wCCMs can also be hierarchically assembled by a confinement process through emulsification in a toluene-in-water emulsion and subsequent evaporation of the solvents. This leads to microparticles with a highly ordered hexagonal close-packed lattice structure [161].

The excellent interfacial activity of patchy wCCMs can be harnessed for the efficient compatibilization of polymer blends, as reported for solvent-cast PS/PMMA (80/20 *w*/*w*) blends [162]. In this work, SEM triblock terpolymers were non-covalently grafted onto the surface of multiwalled CNTs, in order to obtain temperature-stable hybrid compatibilizers with a patchy PS/PMMA corona (patchy CNTs, Figure 6d). The performance of these hybrid compatibilizers was studied depending on their weight fraction, revealing that an increasing filler content considerably reduced the size of the PMMA droplets (minority component) in the blends down to 0.13 µm^2^ for the blend with 9 wt.% patchy CNTs (Figure 9b,c). Remarkably, the obtained PMMA domain areas were significantly lower compared to that achieved by using Janus cylinders (*L* = 2.3 µm, biphasic PS/PMMA corona) as compatibilizers, resulting in domain areas of 10.2 µm^2^ and 1.77 µm^2^ for 5 wt.% and 10 wt.% Janus cylinders (PS/PMMA = 80/20 *w*/*w*), respectively [163]. In addition, the TEM image taken at higher magnification (inset of Figure 9b) shows that well-dispersed patchy CNTs are not only located at the PS/PMMA interface, but are also homogeneously distributed in the PS and PMMA phase. The homogeneous distribution of the patchy CNTs, together with their superior compatibilizing efficiency, can be again attributed to the unique feature of the patchy corona, being able to adapt to their surroundings (PS/PMMA interface, or neat PS and PMMA phases) by selective collapse/expansion of the corona blocks.

### 5.2. Nanoparticle Templates/Hybrids

Metal and metal oxide NPs are highly attractive materials for a multitude of applications, such as optics, medicine, electronics, or catalysis, originating from their unique optical properties and high surface-to-volume ratio [164,165,166,167,168,169,170,171]. However, the high surface area is an ambivalent feature, as it is useful, for example, in catalysis, but considerably limits the overall stability of NP dispersions, due to agglomeration and Ostwald ripening. Here, the stabilization of NPs with ligands has proven to be a convenient solution to overcome this substantial drawback [172,173,174,175]. Another highly efficient method is the use of micellar nanostructures to selectively embed the NPs within functional surface compartments, which not only act as ligands for the NPs, but also keep the NPs’ surface accessible and inhibits agglomeration due to spatial separation [38,117,118,127,137,176,177].

**Figure 10:**
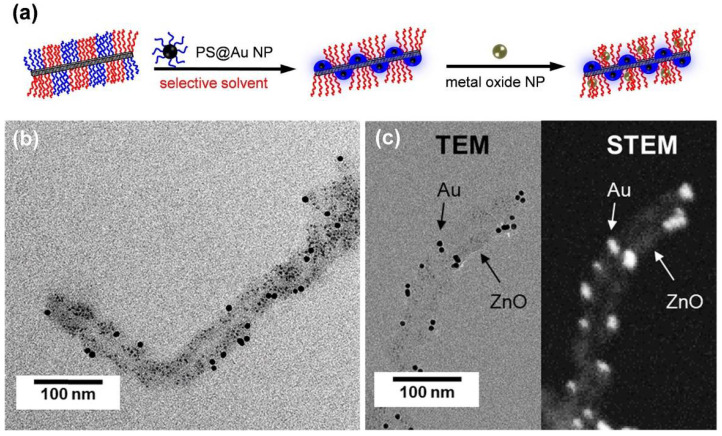
(**a**) Schematic depiction of the regio-selective, binary loading of patchy SEDMA wCCMs with PS-stabilized gold (Au) NPs and zinc oxide (ZnO) NPs, respectively (PS is displayed in blue, PE in black, and PDMA in red). (**b**) TEM image of S_415_E_830_DMA_420_ wCCMs binary-loaded with Au and ZnO NPs. (**c**) Bright-field (left) and high-angle annular dark-field scanning transmission electron microscopy (HAADF-STEM, right) images, clearly revealing the binary loading with two different NP types. Reproduced and adapted from [138] with permission of RSC.

In particular, patchy wCCMs, with their well-defined, alternating segmented coronas, have been shown to be versatile NP templates, and even allow the regio-selective incorporation of two different NP types, since the chemistry of the two corona-forming blocks can be tailored to the specific needs of the respective NP [137,138]. In order to obtain these binary-loaded hybrid materials, based on patchy PS–*b*–PE–*b*–poly(dimethylaminoethyl methacrylamide) (SEDMA) wCCMs, a two-step procedure for the selective decoration of the patches with NPs was developed (Figure 10a). In the first step, preformed, PS-stabilized gold NPs were mixed with a dispersion of the functional patchy wCCMs, followed by the addition of acetone as a selective solvent for the PDMA block, resulting in a collapse of the PS chains. Due to selective interactions of the PS corona block and the PS-stabilized gold NPs, the NPs were enclosed within the PS patches upon collapse of the PS chains. In the following step, preformed, acetate-stabilized zinc oxide NPs were incorporated in the functional patches by a ligand exchange method. Intrinsic staining provided by the inorganic NPs facilitated an examination of the resulting structures via TEM (Figure 10b,c). The different types of NPs are clearly discernible by their different diameters (*D*; *D*_gold NP_ = 7.9 nm, *D*_zinc oxide NP_ = 2.7 nm) and the contrast (heavy metals generate a higher contrast in TEM compared to transition metal oxides). Interestingly, despite the small size of the corona patches (<20 nm), it seems that more than one NP per patch is observable. This might be attributed to the extremely small size of the chosen inorganic NPs (<10 nm).

The selective functionalization of surface-compartmentalized polymeric micelles with inorganic NPs was also shown for PFS-containing triblock co-micelles, featuring a quaternized P2VP corona in the middle [117,118]. Through electrostatic interactions, the middle block was selectively loaded with mercaptoacetic acid-stabilized gold NPs, PbS quantum dots and dextran–magnetite NPs, demonstrating the versatility of block co-micelles as NP templates. Furthermore, NP hybrid materials with block co-micelles derived from co-assembly of diblock copolymers were reported. Here, the spatially confined incorporation of platinum NPs and CdSe quantum dots was enabled by selective interactions with functional corona patches [146,178].

### 5.3. Heterogeneous Catalysis

As mentioned in the previous section, an application of noble metal and metal oxide NPs in catalysis is highly desirable, because of the high catalytically active surface area of the employed NPs. However, ligands, which are needed for stabilization of the NPs, might inhibit the superior catalytic activity of the NPs by blocking the surface. Even for tailor-made ligands, this is a distinct drawback, since these materials are usually hard to recover after usage. A separation of the catalytically active species from the reaction medium is challenging and expensive. Immobilizing the catalytically active NPs on suitable supports (e.g., inorganic, polymeric) solves this problem of recoverability, while preserving the activity and accessibility of the NPs’ surface [179,180,181,182,183,184,185]. Nonetheless, agglomeration of the inorganic NPs on the surface of the heterogeneous supports can occur if the NPs are insufficiently confined, resulting in a significant loss of activity over several consecutive catalysis cycles.

**Figure 11:**
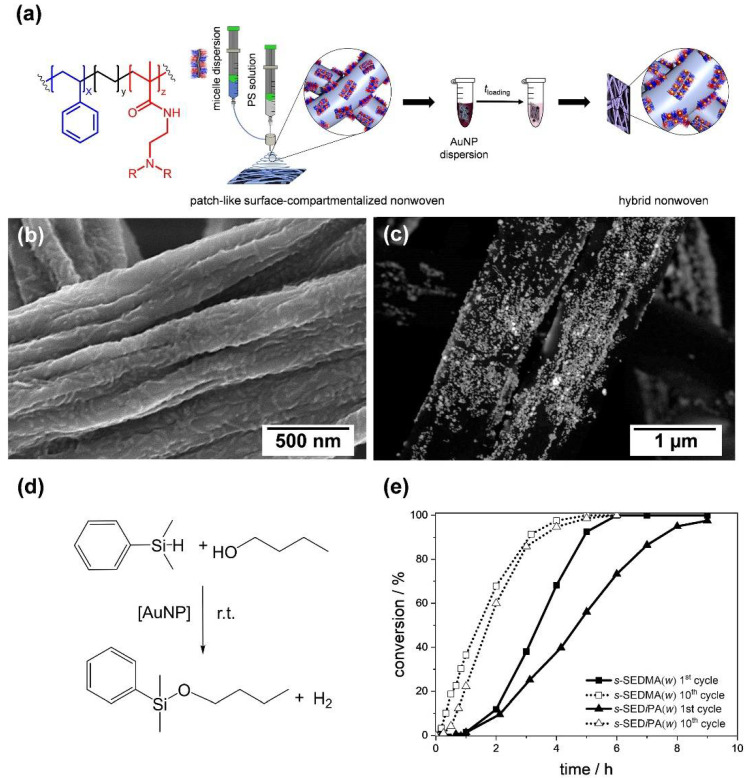
(**a**) Catalytically active, hybrid nonwovens prepared by a combination of bottom-up (CDSA) and top-down (coaxial electrospinning) approaches. In the first step, patchy nonwovens were prepared by decorating a PS nonwoven with functional, patchy PS–*b*–PE–*b*–poly(dialkylaminoethyl methacrylamide) wCCMs by coaxial electrospinning (PS patches are depicted in blue and the functional, tertiary amino group containing patches in red). Subsequently, the patchy nonwovens were loaded with citrate-stabilized Au NPs via a ligand exchange process (citrate against tertiary amino groups in functional patches). (**b**) Scanning electron microscopy images of a patchy nonwoven (based on S_415_E_830_DMA_420_ wCCMs) before and (**c**) after loading with Au NPs (back-scattered electron detector). (**d**) Au NP-catalyzed alcoholysis of dimethylphenylsilane in *n*-butanol. (**e**) Kinetics of the Au NP-catalyzed alcoholysis of dimethylphenylsilane in *n*-butanol, employing patchy hybrid nonwovens as catalysts (D*i*PA = poly(di*iso*propylaminoethyl methacrylamide). Reproduced from [186] with permission from RSC.

The highly regular, alternating arrangement of the corona compartments in patchy wCCMs allows us to efficiently confine inorganic NPs. However, these micellar templates have to be immobilized on a solid support, which provides high accessibility of the reactants to the catalytically active NPs and easy recovery in order to harness these structures for heterogeneous catalysis. This issue was overcome by coating different patchy PS–*b*–PE–*b*–poly(dialkylaminoethyl methacrylamide) wCCMs onto the surface of PS nonwovens by means of coaxial electrospinning (Figure 11a,b) [186,187]. The resulting patchy nonwovens were loaded with gold NPs through a simple dip-coating process (Figure 11c), which was driven by a ligand exchange reaction. The hybrid nonwovens were successfully applied as catalysts for the alcoholysis of dimethylphenylsilane (Figure 11d) at room temperature, showing a comparable or even higher catalytic activity than other supports reported before [188,189,190,191,192,193]. Moreover, the employed patchy hybrid nonwovens were easily recoverable from the reaction medium and reusable in at least 10 consecutive catalysis cycles.

Since this system offers different possibilities to tune the catalytic activity, an in-depth study on the influence of the patch size and chemistry on the reaction kinetics was conducted. Here, an extended first-order kinetics model was employed, which includes the induction periods observed in the catalytic alcoholysis of dimethylphenylsilane in *n*-butanol. This study revealed a strong dependence on the accessibility of the reactants to the gold NPs’ surface, being mainly controlled by the swellability of the functional patches in *n*-butanol. The latter depends on both patch chemistry, i.e., poly(*N*,*N*-dimethylaminoethyl methacrylamide) (PDMA, more hydrophilic) vs. poly(*N*,*N*-di*iso*propylaminoethyl methacrylamide) (PD*i*PA, more hydrophobic) patches, as well as size. As a result, significantly longer induction (*t*_ind_) and reaction (*t*_R_) times were observed for the first catalysis cycles compared to the tenth cycles (Figure 11e). Nonwovens with more polar PDMA patches were the most efficient in NP stabilization (prevention of agglomeration), but showed a significantly lower *t*_R_ in the first catalysis cycle, due to a strong interaction with the gold NPs’ surface. Thus, precise tuning of the patch size and chemistry is needed to optimize the catalysts performance. However, the modular design of the patchy hybrid nonwovens enables a facile adaption to the needs of different catalysis systems—for example, by an exchange of the support material or by varying the type of NPs. Moreover, it is possible to render the functionalized patches thermo-responsive [194], which opens access to catalytic reactions regulated by an inherent temperature control.

## 6. Conclusions and Outlook

From a conceptual point of view, several strategies exist for the production of patchy micelles with crystalline cores, such as CDSA of triblock terpolymers with crystallizable middle blocks, miktoarm stars, or the co-assembly of diblock copolymers with a common crystallizable block but different corona-forming blocks. However, so far, patchy micelles have been reported only for BCPs with PE or PFS as crystallizable blocks, despite the fact that a large variety of crystallizable polymer blocks has already been utilized in CDSA. Here, ring-opening polymerization of lactones or lactides, in combination with controlled radical polymerization techniques, might be another promising alternative, as BCPs based on PCL or PLLA as crystallizable blocks are readily accessible. Moreover, PFS could be replaced by ruthenocene-based BCPs, which show a higher degree of crystallinity but are less studied for CDSA. Finally, patchy micelles could be derived from the simultaneous heteroepitaxial growth of two crystallizable di- or triblock copolymers bearing different core- and corona-forming blocks, inducing segmentation within the core as well as in the corona.

The alternating arrangement of the corona patches emerges as an excellent feature for the stabilization and confinement of metal and metal oxide nanoparticles, opening applications in heterogeneous catalysis. Yet this has been shown only for the gold nanoparticle-catalyzed alcoholysis of silanes, and it is anticipated that this concept can be transferred to other relevant catalytic processes like heterogeneous hydrogenation. By incorporating different nanoparticle types, even cascade reactions might be realizable. Most interestingly, the interfacial activity of patchy, worm-like (or cylindrical) micelles is equivalent to that of Janus micelles, the latter being, however, more difficult to produce. Thus, patchy micelles might be utilized in interfacial catalysis, as well as in the efficient stabilization of emulsions or compatibilization of polymer blends.

## Figures and Tables

**Table 1:** Overview of self-assembly concepts for patchy micelles with a crystalline core.

Self-Assembly Concept	Employed BCPs	Special Feature	Reference
**CDSA of triblock terpolymers**			
Linear triblock terpolymers	PS–*b*–PE–*b*–PMMA	Control over micelle morphology, length control through seeded growth, co-crystallization with PS–*b*–PE–*b*–PS	[93,94,134,135,136]
	PS–*b*–PE–*b*–PDxA^1^	Functional groups for NP incorporation	[137,138]
	PS–*b*–PFS–*b*–PMMA	Control over patch size, co-crystallization with diblock co-polymers of varying PS and PMMA block lengths	[139]
	PS–*b*–PFS–*b*–PMVS, PI–*b*–PFS–*b*–PMMA	Length control through seeded growth	[140]
Star-shaped triblock terpolymers	*µ*-SIF	Seeded growth, block co-micelles with patchy *µ*-SIF outer blocks, and middle block based on PFS-*b*-PDMS	[141]
Non-covalent grafting on carbon nanotubes	PS–*b*–PE–*b*–PMMA	Temperature-stable patchy hybrid materials	[142]
**Co-assembly of diblock copolymers**			
Sterically demanding co-unimers	PFS–*b*–PMVS, PFS–*b*–PMVS(C18)^2^	Gradual coassembly of linear and brush-type BCPs	[143]
Strong difference in Flory–Huggins interaction parameters of corona chains	PFS–*b*–PDMS, PFS–*b*–PMVS, PFS–*b*–PI	Different patch arrangements accessible (helical, hemispherical)	[144]
Manipulation of the epitaxial growth rate or the critical dissolution temperature	PFS–*b*–P2VP, PFS–*b*–PNiPAM, PFS–*b*–P2VPQ^3^	Patchy or blocky structures accessible	[80,145]
Addition of crystallizable homopolymer, heating–cooling–aging approach	PFS, PFS–*b*–PDMS, PFS–*b*–PI, PFS–*b*–PMVS, PFS–*b*–P2VP	PFS crystal fragments serve as seeds, patchy or blocky structures, easy up-scaling	[146]

^1^ PDxA: poly(*N,N*-dialkylaminoethyl methacrylamide). ^2^ PMVS block alkylated by C18 alkyl chains. ^3^ Quaternized P2VP.

## Data Availability

Not applicable.

## References

[1] Tritschler U., Pearce S., Gwyther J., Whittell G.R., Manners I. (2017). 50th Anniversary Perspective: Functional Nanoparticles from the Solution Self-Assembly of Block Copolymers. Macromolecules.

[2] Du J., O’Reilly R.K. (2011). Anisotropic particles with patchy, multicompartment and Janus architectures: Preparation and application. Chem. Soc. Rev..

[3] Wyman I.W., Liu G. (2013). Micellar structures of linear triblock terpolymers: Three blocks but many possibilities. Polymer.

[4] Schacher F.H., Rupar P.A., Manners I. (2012). Functional Block Copolymers: Nanostructured Materials with Emerging Applications. Angew. Chem. Int. Ed..

[5] Matyjaszewski K., Möller M. (2012). Polymer Science: A Comprehensive Reference.

[6] Feng H., Lu X., Wang W., Kang N.-G., Mays J.W. (2017). Block Copolymers: Synthesis, Self-Assembly, and Applications. Polymers.

[7] Müller A.H.E., Matyjaszewski K. (2009). Controlled and Living Polymerizations.

[8] Jennings J., He G., Howdle S.M., Zetterlund P.B. (2016). Block copolymer synthesis by controlled/living radical polymerisation in heterogeneous systems. Chem. Soc. Rev..

[9] Hadjichristidis N., Pitsikalis M., Iatrou H., Abetz V. (2005). Synthesis of Block Copolymers. Block Copolymers I. Advances in Polymer Science.

[10] Lutz J., Laschewsky A. (2005). Multicompartment Micelles: Has the Long-Standing Dream Become a Reality?. Macromol. Chem. Phys..

[11] Laschewsky A. (2003). Polymerized micelles with compartments. Curr. Opin. Colloid Interface Sci..

[12] Gröschel A.H., Walther A., Löbling T.I., Schacher F.H., Schmalz H., Müller A.H.E. (2013). Guided hierarchical co-assembly of soft patchy nanoparticles. Nature.

[13] Li W., Palis H., Mérindol R., Majimel J., Ravaine S., Duguet E. (2020). Colloidal molecules and patchy particles: Complementary concepts, synthesis and self-assembly. Chem. Soc. Rev..

[14] Lunn D.J., Finnegan J.R., Manners I. (2015). Self-assembly of “patchy” nanoparticles: A versatile approach to functional hierarchical materials. Chem. Sci..

[15] Zhang K., Jiang M., Chen D. (2012). Self-assembly of particles—The regulatory role of particle flexibility. Prog. Polym. Sci..

[16] Löbling T.I., Ikkala O., Gröschel A.H., Müller A.H.E. (2016). Controlling Multicompartment Morphologies Using Solvent Conditions and Chemical Modification. ACS Macro Lett..

[17] Fang B., Walther A., Wolf A., Xu Y., Yuan J., Müller A.H.E. (2009). Undulated Multicompartment Cylinders by the Controlled and Directed Stacking of Polymer Micelles with a Compartmentalized Corona. Angew. Chem. Int. Ed..

[18] Lee S., Jang S., Kim K., Jeon J., Kim S.-S., Sohn B.-H. (2016). Branched and crosslinked supracolloidal chains with diblock copolymer micelles having three well-defined patches. Chem. Commun..

[19] Walther A., Müller A.H.E. (2009). Formation of hydrophobic bridges between multicompartment micelles of miktoarm star terpolymers in water. Chem. Commun..

[20] Kong W., Jiang W., Zhu Y., Li B. (2012). Highly Symmetric Patchy Multicompartment Nanoparticles from the Self-Assembly of ABC Linear Terpolymers in C-Selective Solvents. Langmuir.

[21] Kim K., Jang S., Jeon J., Kang D., Sohn B.-H. (2018). Fluorescent Supracolloidal Chains of Patchy Micelles of Diblock Copolymers Functionalized with Fluorophores. Langmuir.

[22] Nghiem T., Chakroun R., Janoszka N., Chen C., Klein K., Wong C.K., Gröschel A.H. (2020). pH-Controlled Hierarchical Assembly/Disassembly of Multicompartment Micelles in Water. Macromol. Rapid Commun..

[23] Skrabania K., Berlepsch H.V., Böttcher C., Laschewsky A. (2010). Synthesis of Ternary, Hydrophilic−Lipophilic−Fluorophilic Block Copolymers by Consecutive RAFT Polymerizations and Their Self-Assembly into Multicompartment Micelles. Macromolecules.

[24] Löbling T.I., Borisov O., Haataja J.S., Ikkala O., Gröschel A.H., Müller A.H.E. (2016). Rational design of ABC triblock terpolymer solution nanostructures with controlled patch morphology. Nat. Commun..

[25] Nghiem T.-L., Löbling T.I., Gröschel A.H. (2017). Supracolloidal chains of patchy micelles in water. Polym. Chem..

[26] Moughton A.O., Hillmyer M.A., Lodge T.P. (2012). Multicompartment Block Polymer Micelles. Macromolecules.

[27] Wang L., Lin J. (2011). Discovering multicore micelles: Insights into the self-assembly of linear ABC terpolymers in midblock-selective solvents. Soft Matter.

[28] Gröschel A.H., Müller A.H.E. (2015). Self-assembly concepts for multicompartment nanostructures. Nanoscale.

[29] Wong C.K., Qiang X., Müller A.H.E., Gröschel A.H. (2020). Self-Assembly of block copolymers into internally ordered microparticles. Prog. Polym. Sci..

[30] Pelras T., Mahon C.S., Müllner M. (2018). Synthesis and Applications of Compartmentalised Molecular Polymer Brushes. Angew. Chem. Int. Ed..

[31] Nayanathara U., Kermaniyan S.S., Such G.K. (2020). Multicompartment Polymeric Nanocarriers for Biomedical Applications. Macromol. Rapid Commun..

[32] Marschelke C., Fery A., Synytska A. (2020). Janus particles: From concepts to environmentally friendly materials and sustainable applications. Colloid Polym. Sci..

[33] Fan X., Yang J., Loh X.J., Li Z. (2019). Polymeric Janus Nanoparticles: Recent Advances in Synthetic Strategies, Materials Properties, and Applications. Macromol. Rapid Commun..

[34] Agrawal G., Agrawal R. (2019). Janus Nanoparticles: Recent Advances in Their Interfacial and Biomedical Applications. ACS Appl. Nano Mater..

[35] Zhang J., Grzybowski B.A., Granick S. (2017). Janus Particle Synthesis, Assembly, and Application. Langmuir.

[36] Deng R., Liang F., Zhu J., Yang Z. (2016). Recent advances in the synthesis of Janus nanomaterials of block copolymers. Mater. Chem. Front..

[37] Pang X., Wan C., Wang M., Lin Z. (2014). Strictly Biphasic Soft and Hard Janus Structures: Synthesis, Properties, and Applications. Angew. Chem. Int. Ed..

[38] Walther A., Müller A.H.E. (2013). Janus Particles: Synthesis, Self-Assembly, Physical Properties, and Applications. Chem. Rev..

[39] Hu J., Zhou S., Sun Y., Fang X., Wu L. (2012). Fabrication, properties and applications of Janus particles. Chem. Soc. Rev..

[40] Loget G., Kuhn A. (2012). Bulk synthesis of Janus objects and asymmetric patchy particles. J. Mater. Chem..

[41] Wurm F., Kilbinger A.F.M. (2009). Polymeric Janus Particles. Angew. Chem. Int. Ed..

[42] Dou H., Liu G., Dupont J., Hong L. (2010). Triblock terpolymer helices self-assembled under special solvation conditions. Soft Matter.

[43] Dupont J., Liu G., Niihara K.-I., Kimoto R., Jinnai H. (2009). Self-Assembled ABC Triblock Copolymer Double and Triple Helices. Angew. Chem. Int. Ed..

[44] Hoppenbrouwers E., Li Z., Liu G. (2003). Triblock Nanospheres with Amphiphilic Coronal Chains. Macromolecules.

[45] Hu J., Njikang G., Liu G. (2008). Twisted ABC Triblock Copolymer Cylinders with Segregated A and C Coronal Chains. Macromolecules.

[46] Liu X., Ding Y., Liu J., Lin S., Zhuang Q. (2019). Evolution in the morphological behaviour of a series of fluorine-containing ABC miktoarm star terpolymers. Eur. Polym. J..

[47] Njikang G., Han D., Wang J., Liu G. (2008). ABC Triblock Copolymer Micelle-Like Aggregates in Selective Solvents for A and C. Macromolecules.

[48] Zhang W., He J.X., Liu Q., Ke G.Q., Dong X. (2014). Synthesis of Block Terpolymer PS-PDMAEMA-PMMA via ATRP and its Self-Assembly in Selective Solvents. Adv. Mater. Res..

[49] Kuo S.-W., Tung P.-H., Lai C.-L., Jeong K.-U., Chang F.-C. (2007). Supramolecular Micellization of Diblock Copolymer Mixtures Mediated by Hydrogen Bonding for the Observation of Separated Coil and Chain Aggregation in Common Solvents. Macromol. Rapid Commun..

[50] Kuo S.-W., Tung P.-H., Chang F.-C. (2009). Hydrogen bond mediated supramolecular micellization of diblock copolymer mixture in common solvents. Eur. Polym. J..

[51] Voets I.K., De Keizer A., Leermakers F.A., Debuigne A., Jerôme R., Detrembleur C., Stuart M.A.C. (2009). Electrostatic hierarchical co-assembly in aqueous solutions of two oppositely charged double hydrophilic diblock copolymers. Eur. Polym. J..

[52] Lopresti C., Massignani M., Fernyhough C., Blanazs A., Ryan A.J., Madsen J., Warren N.J., Armes S.P., Lewis A.L., Chirasatitsin S. (2011). Controlling Polymersome Surface Topology at the Nanoscale by Membrane Confined Polymer/Polymer Phase Separation. ACS Nano.

[53] Hu J., Liu G. (2005). Chain Mixing and Segregation in B−C and C−D Diblock Copolymer Micelles. Macromolecules.

[54] Srinivas G., Pitera J.W. (2008). Soft Patchy Nanoparticles from Solution-Phase Self-Assembly of Binary Diblock Copolymers. Nano Lett..

[55] Zheng R., Liu G., Yan X. (2005). Polymer Nano- and Microspheres with Bumpy and Chain-Segregated Surfaces. J. Am. Chem. Soc..

[56] Christian D.A., Tian A., Ellenbroek W.G., Levental I., Rajagopal K., Janmey P.A., Liu A.J., Baumgart T., Discher D.E. (2009). Spotted vesicles, striped micelles and Janus assemblies induced by ligand binding. Nat. Mater..

[57] Hsu H.-P., Paul W., Binder K. (2007). One- and Two-Component Bottle-Brush Polymers: Simulations Compared to Theoretical Predictions. Macromol. Theory Simul..

[58] De Jong J., ten Brinke G. (2004). Conformational Aspects and Intramolecular Phase Separation of Alternating Copolymacromonomers: A Computer Simulation Study. Macromol. Theory Simul..

[59] Stepanyan R., Subbotin A., ten Brinke G. (2002). Comb Copolymer Brush with Chemically Different Side Chains. Macromolecules.

[60] Theodorakis P.E., Paul W., Binder K. (2010). Interplay between Chain Collapse and Microphase Separation in Bottle-Brush Polymers with Two Types of Side Chains. Macromolecules.

[61] Hsu H.-P., Paul W., Binder K. (2006). Intramolecular phase separation of copolymer “bottle brushes”: No sharp phase transition but a tunable length scale. EPL Europhys. Lett..

[62] He W.-N., Xu J.-T. (2012). Crystallization assisted self-assembly of semicrystalline block copolymers. Prog. Polym. Sci..

[63] Ganda S., Stenzel M.H. (2020). Concepts, fabrication methods and applications of living crystallization-driven self-assembly of block copolymers. Prog. Polym. Sci..

[64] Hailes R.L.N., Oliver A.M., Gwyther J., Whittell G.R., Manners I. (2016). Polyferrocenylsilanes: Synthesis, properties, and applications. Chem. Soc. Rev..

[65] Wang X., Guerin G., Wang H., Wang Y., Manners I., Winnik M.A. (2007). Cylindrical Block Copolymer Micelles and Co-Micelles of Controlled Length and Architecture. Science.

[66] Gilroy J.B., Gädt T., Whittell G.R., Chabanne L., Mitchels J.M., Richardson R.M., Winnik M.A., Manners I. (2010). Monodisperse cylindrical micelles by crystallization-driven living self-assembly. Nat. Chem..

[67] MacFarlane L., Zhao C., Cai J., Qiu H., Manners I. (2021). Emerging applications for living crystallization-driven self-assembly. Chem. Sci..

[68] Schmelz J., Schedl A.E., Steinlein C., Manners I., Schmalz H. (2012). Length Control and Block-Type Architectures in Worm-like Micelles with Polyethylene Cores. J. Am. Chem. Soc..

[69] Qian J., Lu Y., Chia A., Zhang M., Rupar P.A., Gunari N., Walker G.C., Cambridge G., He F., Guerin G. (2013). Self-Seeding in One Dimension: A Route to Uniform Fiber-like Nanostructures from Block Copolymers with a Crystallizable Core-Forming Block. ACS Nano.

[70] Qian J., Guerin G., Lu Y., Cambridge G., Manners I., Winnik M.A. (2011). Self-Seeding in One Dimension: An Approach To Control the Length of Fiberlike Polyisoprene-Polyferrocenylsilane Block Copolymer Micelles. Angew. Chem. Int. Ed..

[71] Li X., Jin B., Gao Y., Hayward D.W., Winnik M.A., Luo Y., Manners I. (2016). Monodisperse Cylindrical Micelles of Controlled Length with a Liquid-Crystalline Perfluorinated Core by 1D “Self-Seeding”. Angew. Chem. Int. Ed..

[72] Boott C.E., Gwyther J., Harniman R.L., Hayward D.W., Manners I. (2017). Scalable and uniform 1D nanoparticles by synchronous polymerization, crystallization and self-assembly. Nat. Chem..

[73] Oliver A.M., Gwyther J., Boott C.E., Davis S., Pearce S., Manners I. (2018). Scalable Fiber-like Micelles and Block Co-micelles by Polymerization-Induced Crystallization-Driven Self-Assembly. J. Am. Chem. Soc..

[74] Sha Y., Rahman A., Zhu T., Cha Y., McAlister C.W., Tang C. (2019). ROMPI-CDSA: Ring-opening metathesis polymerization-induced crystallization-driven self-assembly of metallo-block copolymers. Chem. Sci..

[75] Shin S., Menk F., Kim Y., Lim J., Char K., Zentel R., Choi T.-L. (2018). Living Light-Induced Crystallization-Driven Self-Assembly for Rapid Preparation of Semiconducting Nanofibers. J. Am. Chem. Soc..

[76] Qiu H., Gao Y., Du V.A., Harniman R., Winnik M.A., Manners I. (2015). Branched Micelles by Living Crystallization-Driven Block Copolymer Self-Assembly under Kinetic Control. J. Am. Chem. Soc..

[77] Hudson Z.M., Lunn D.J., Winnik M.A., Manners I. (2014). Colour-tunable fluorescent multiblock micelles. Nat. Commun..

[78] Jin X.-H., Price M.B., Finnegan J.R., Boott C.E., Richter J.M., Rao A., Menke S.M., Friend R.H., Whittell G.R., Manners I. (2018). Long-range exciton transport in conjugated polymer nanofibers prepared by seeded growth. Science.

[79] Rupar P.A., Chabanne L., Winnik M.A., Manners I. (2012). Non-Centrosymmetric Cylindrical Micelles by Unidirectional Growth. Science.

[80] Xu J., Zhou H., Yu Q., Guerin G., Manners I., Winnik M.A. (2019). Synergistic self-seeding in one-dimension: A route to patchy and block comicelles with uniform and controllable length. Chem. Sci..

[81] He X., He Y., Hsiao M.-S., Harniman R.L., Pearce S., Winnik M.A., Manners I. (2017). Complex and Hierarchical 2D Assemblies via Crystallization-Driven Self-Assembly of Poly(L-lactide) Homopolymers with Charged Termini. J. Am. Chem. Soc..

[82] He X., Hsiao M.-S., Boott C.E., Harniman R.L., Nazemi A., Li X., Winnik M.A., Manners I. (2017). Two-dimensional assemblies from crystallizable homopolymers with charged termini. Nat. Mater..

[83] Nazemi A., He X., Macfarlane L.R., Harniman R.L., Hsiao M.-S., Winnik M.A., Faul C.F.J., Manners I. (2017). Uniform “Patchy” Platelets by Seeded Heteroepitaxial Growth of Crystallizable Polymer Blends in Two Dimensions. J. Am. Chem. Soc..

[84] Pearce S., He X., Hsiao M.-S., Harniman R.L., Macfarlane L.R., Manners I. (2019). Uniform, High-Aspect-Ratio, and Patchy 2D Platelets by Living Crystallization-Driven Self-Assembly of Crystallizable Poly(ferrocenyldimethylsilane)-Based Homopolymers with Hydrophilic Charged Termini. Macromolecules.

[85] Qiu H., Gao Y., Boott C.E., Gould O.E.C., Harniman R.L., Miles M.J., Webb S.E.D., Winnik M.A., Manners I. (2016). Uniform patchy and hollow rectangular platelet micelles from crystallizable polymer blends. Science.

[86] Inam M., Cambridge G., Pitto-Barry A., Laker Z.P.L., Wilson N.R., Mathers R.T., Dove A.P., O’Reilly R.K. (2017). 1D vs. 2D shape selectivity in the crystallization-driven self-assembly of polylactide block copolymers. Chem. Sci..

[87] Gould O.E., Qiu H., Lunn D.J., Rowden J., Harniman R.L., Hudson Z.M., Winnik M.A., Miles M.J., Manners I. (2015). Transformation and patterning of supermicelles using dynamic holographic assembly. Nat. Commun..

[88] Qiu H., Hudson Z.M., Winnik M.A., Manners I. (2015). Multidimensional hierarchical self-assembly of amphiphilic cylindrical block comicelles. Science.

[89] Gädt T., Ieong N.S., Cambridge G., Winnik M.A., Manners I. (2009). Complex and hierarchical micelle architectures from diblock copolymers using living, crystallization-driven polymerizations. Nat. Mater..

[90] Hudson Z.M., Boott C.E., Robinson M.E., Rupar P.A., Winnik M.A., Manners I. (2014). Tailored hierarchical micelle architectures using living crystallization-driven self-assembly in two dimensions. Nat. Chem..

[91] Dou H., Li M., Qiao Y., Harniman R., Li X., Boott C.E., Mann S., Manners I. (2017). Higher-order assembly of crystalline cylindrical micelles into membrane-extendable colloidosomes. Nat. Commun..

[92] Fan B., Liu L., Li J.-H., Ke X.-X., Xu J.-T., Du B.-Y., Fan Z.-Q. (2015). Crystallization-driven one-dimensional self-assembly of polyethylene-*b*-poly(*tert*-butylacrylate) diblock copolymers in DMF: Effects of crystallization temperature and the corona-forming block. Soft Matter.

[93] Schmalz H., Schmelz J., Drechsler M., Yuan J., Walther A., Schweimer K., Mihut A.M. (2008). Thermo-Reversible Formation of Wormlike Micelles with a Microphase-Separated Corona from a Semicrystalline Triblock Terpolymer. Macromolecules.

[94] Schmelz J., Karg M., Hellweg T., Schmalz H. (2011). General Pathway toward Crystalline-Core Micelles with Tunable Morphology and Corona Segregation. ACS Nano.

[95] Xiong H., Chen C.-K., Lee K., Van Horn R.M., Liu Z., Ren B., Quirk R.P., Thomas E.L., Lotz B., Ho R.-M. (2011). Scrolled Polymer Single Crystals Driven by Unbalanced Surface Stresses: Rational Design and Experimental Evidence. Macromolecules.

[96] Coe Z., Weems A., Dove A.P., O’Reilly R.K. (2019). Synthesis of Monodisperse Cylindrical Nanoparticles via Crystallization-driven Self-assembly of Biodegradable Block Copolymers. J. Vis. Exp..

[97] Arno M.C., Inam M., Coe Z., Cambridge G., MacDougall L.J., Keogh R., Dove A.P., O’Reilly R.K. (2017). Precision Epitaxy for Aqueous 1D and 2D Poly(ε-caprolactone) Assemblies. J. Am. Chem. Soc..

[98] Petzetakis N., Dove A.P., O’Reilly R.K. (2011). Cylindrical micelles from the living crystallization-driven self-assembly of poly(lactide)-containing block copolymers. Chem. Sci..

[99] Arno M.C., Inam M., Weems A.C., Li Z., Binch A.L.A., Platt C.I., Richardson S.M., Hoyland J.A., Dove A.P., O’Reilly R.K. (2020). Exploiting the role of nanoparticle shape in enhancing hydrogel adhesive and mechanical properties. Nat. Commun..

[100] Yu W., Inam M., Jones J.R., Dove A.P., O’Reilly R.K. (2017). Understanding the CDSA of poly(lactide) containing triblock copolymers. Polym. Chem..

[101] Tong Z., Su Y., Jiang Y., Xie Y., Chen S., O’Reilly R.K. (2021). Spatially Restricted Templated Growth of Poly(ε-caprolactone) from Carbon Nanotubes by Crystallization-Driven Self-Assembly. Macromolecules.

[102] Finnegan J.R., He X., Street S.T.G., Garcia-Hernandez J.D., Hayward D.W., Harniman R.L., Richardson R.M., Whittell G.R., Manners I. (2018). Extending the Scope of “Living” Crystallization-Driven Self-Assembly: Well-Defined 1D Micelles and Block Comicelles from Crystallizable Polycarbonate Block Copolymers. J. Am. Chem. Soc..

[103] Rudolph T., von der Lühe M., Hartlieb M., Norsic S., Schubert U.S., Boisson C., D’Agosto F., Schacher F.H. (2015). Toward Anisotropic Hybrid Materials: Directional Crystallization of Amphiphilic Polyoxazoline-Based Triblock Terpolymers. ACS Nano.

[104] Finnegan J., Pilkington E., Alt K., Rahim A., Kent S.J., Davis T.P., Kempe K. (2021). Stealth Nanorods via the Aqueous Living Crystallisation-Driven Self-Assembly of Poly(2-oxazoline)s. Chem. Sci..

[105] Shaikh H., Jin X.-H., Harniman R.L., Richardson R.M., Whittell G.R., Manners I. (2020). Solid-State Donor-Acceptor Coaxial Heterojunction Nanowires via Living Crystallization-Driven Self-Assembly. J. Am. Chem. Soc..

[106] Folgado E., Mayor M., Cot D., Ramonda M., Godiard F., Ladmiral V., Semsarilar M. (2021). Preparation of well-defined 2D-lenticular aggregates by self-assembly of PNIPAM-*b*-PVDF amphiphilic diblock copolymers in solution. Polym. Chem..

[107] Kang L., Chao A., Zhang M., Yu T., Wang J., Wang Q., Yu H., Jiang N., Zhang D. (2021). Modulating the Molecular Geometry and Solution Self-Assembly of Amphiphilic Polypeptoid Block Copolymers by Side Chain Branching Pattern. J. Am. Chem. Soc..

[108] Wei Y., Liu F., Li M., Li Z., Sun J. (2021). Dimension control on self-assembly of a crystalline core-forming polypeptoid block copolymer: 1D nanofibers versus 2D nanosheets. Polym. Chem..

[109] Kynaston E.L., Nazemi A., MacFarlane L.R., Whittell G.R., Faul C.F.J., Manners I. (2018). Uniform Polyselenophene Block Copolymer Fiberlike Micelles and Block Co-micelles via Living Crystallization-Driven Self-Assembly. Macromolecules.

[110] Kim Y.-J., Cho C.-H., Paek K., Jo M., Park M.-K., Lee N.-E., Kim Y.-J., Kim B.J., Lee E. (2014). Precise Control of Quantum Dot Location within the P3HT-*b*-P2VP/QD Nanowires Formed by Crystallization-Driven 1D Growth of Hybrid Dimeric Seeds. J. Am. Chem. Soc..

[111] Patra S.K., Ahmed R., Whittell G.R., Lunn D.J., Dunphy E.L., Winnik M.A., Manners I. (2011). Cylindrical Micelles of Controlled Length with a π-Conjugated Polythiophene Core via Crystallization-Driven Self-Assembly. J. Am. Chem. Soc..

[112] Li X., Wolanin P.J., MacFarlane L.R., Harniman R.L., Qian J., Gould O.E.C., Dane T.G., Rudin J., Cryan M.J., Schmaltz T. (2017). Uniform electroactive fibre-like micelle nanowires for organic electronics. Nat. Commun..

[113] MacFarlane L.R., Shaikh H., Garcia-Hernandez J.D., Vespa M., Fukui T., Manners I. (2021). Functional nanoparticles through π-conjugated polymer self-assembly. Nat. Rev. Mater..

[114] Boott C.E., Laine R.F., Mahou P., Finnegan J.R., Leitao E.M., Webb S.E.D., Kaminski C.F., Manners I. (2015). In Situ Visualization of Block Copolymer Self-Assembly in Organic Media by Super-Resolution Fluorescence Microscopy. Chem. Eur. J..

[115] Wittmann J.C., Hodge A.M., Lotz B. (1983). Epitaxial crystallization of polymers onto benzoic acid: Polyethylene and paraffins, aliphatic polyesters, and polyamides. J. Polym. Sci. Polym. Phys. Ed..

[116] Wittmann J.C., Lotz B. (1981). Epitaxial crystallization of polyethylene on organic substrates: A reappraisal of the mode of action of selected nucleating agents. J. Polym. Sci. Polym. Phys. Ed..

[117] Wang H., Lin W., Fritz K.P., Scholes G.D., Winnik M.A., Manners I. (2007). Cylindrical Block Co-Micelles with Spatially Selective Functionalization by Nanoparticles. J. Am. Chem. Soc..

[118] Wang H., Patil A.J., Liu K., Petrov S., Mann S., Winnik M.A., Manners I. (2009). Fabrication of Continuous and Segmented Polymer/Metal Oxide Nanowires Using Cylindrical Micelles and Block Comicelles as Templates. Adv. Mater..

[119] Rupar P.A., Cambridge G., Winnik M.A., Manners I. (2011). Reversible Cross-Linking of Polyisoprene Coronas in Micelles, Block Comicelles, and Hierarchical Micelle Architectures Using Pt(0)–Olefin Coordination. J. Am. Chem. Soc..

[120] Nazemi A., Boott C.E., Lunn D.J., Gwyther J., Hayward D.W., Richardson R.M., Winnik M.A., Manners I. (2016). Monodisperse Cylindrical Micelles and Block Comicelles of Controlled Length in Aqueous Media. J. Am. Chem. Soc..

[121] Qiu H., Cambridge G., Winnik M.A., Manners I. (2013). Multi-Armed Micelles and Block Co-micelles via Crystallization-Driven Self-Assembly with Homopolymer Nanocrystals as Initiators. J. Am. Chem. Soc..

[122] He F., Gädt T., Manners I., Winnik M.A. (2011). Fluorescent “Barcode” Multiblock Co-Micelles via the Living Self-Assembly of Di- and Triblock Copolymers with a Crystalline Core-Forming Metalloblock. J. Am. Chem. Soc..

[123] Street S.T.G., He Y., Jin X.-H., Hodgson L., Verkade P., Manners I. (2020). Cellular uptake and targeting of low dispersity, dual emissive, segmented block copolymer nanofibers. Chem. Sci..

[124] He X., Finnegan J.R., Hayward D.W., MacFarlane L.R., Harniman R.L., Manners I. (2020). Living Crystallization-Driven Self-Assembly of Polymeric Amphiphiles: Low-Dispersity Fiber-like Micelles from Crystallizable Phosphonium-Capped Polycarbonate Homopolymers. Macromolecules.

[125] Qian J., Li X., Lunn D.J., Gwyther J., Hudson Z.M., Kynaston E., Rupar P.A., Winnik M.A., Manners I. (2014). Uniform, High Aspect Ratio Fiber-like Micelles and Block Co-micelles with a Crystalline π-Conjugated Polythiophene Core by Self-Seeding. J. Am. Chem. Soc..

[126] Tao D., Feng C., Cui Y., Yang X., Manners I., Winnik M.A., Huang X. (2017). Monodisperse Fiber-like Micelles of Controlled Length and Composition with an Oligo(*p*-phenylenevinylene) Core via “Living” Crystallization-Driven Self-Assembly. J. Am. Chem. Soc..

[127] Tao D., Wang Z., Huang X., Tian M., Lu G., Manners I., Winnik M.A., Feng C. (2020). Continuous and Segmented Semiconducting Fiber-like Nanostructures with Spatially Selective Functionalization by Living Crystallization-Driven Self-Assembly. Angew. Chem. Int. Ed..

[128] He Y., Eloi J.-C., Harniman R.L., Richardson R.M., Whittell G.R., Mathers R.T., Dove A.P., O’Reilly R.K., Manners I. (2019). Uniform Biodegradable Fiber-Like Micelles and Block Comicelles via “Living” Crystallization-Driven Self-Assembly of Poly(L-lactide) Block Copolymers: The Importance of Reducing Unimer Self-Nucleation via Hydrogen Bond Disruption. J. Am. Chem. Soc..

[129] Lunn D.J., Gould O.E.C., Whittell G.R., Armstrong D.P., Mineart K.P., Winnik M.A., Spontak R.J., Pringle P.G., Manners I. (2016). Microfibres and macroscopic films from the coordination-driven hierarchical self-assembly of cylindrical micelles. Nat. Commun..

[130] Li X., Gao Y., Boott C.E., Hayward D.W., Harniman R., Whittell G.R., Richardson R.M., Winnik M.A., Manners I. (2016). “Cross” Supermicelles via the Hierarchical Assembly of Amphiphilic Cylindrical Triblock Comicelles. J. Am. Chem. Soc..

[131] Qiu H., Russo G., Rupar P.A., Chabanne L., Winnik M.A., Manners I. (2012). Tunable Supermicelle Architectures from the Hierarchical Self-Assembly of Amphiphilic Cylindrical B-A-B Triblock Co-Micelles. Angew. Chem. Int. Ed..

[132] Li X., Gao Y., Boott C.E., Winnik M.A., Manners I. (2015). Non-covalent synthesis of supermicelles with complex architectures using spatially confined hydrogen-bonding interactions. Nat. Commun..

[133] Li X., Gao Y., Harniman R., Winnik M., Manners I. (2016). Hierarchical Assembly of Cylindrical Block Comicelles Mediated by Spatially Confined Hydrogen-Bonding Interactions. J. Am. Chem. Soc..

[134] Schmelz J., Schacher F.H., Schmalz H. (2013). Cylindrical crystalline-core micelles: Pushing the limits of solution self-assembly. Soft Matter.

[135] Rosenfeldt S., Lüdel F., Schulreich C., Hellweg T., Radulescu A., Schmelz J., Schmalz H., Harnau L. (2012). Patchy worm-like micelles: Solution structure studied by small-angle neutron scattering. Phys. Chem. Chem. Phys..

[136] Schmelz J., Schmalz H. (2012). Corona structure on demand: Tailor-made surface compartmentalization in worm-like micelles via random cocrystallization. Polymer.

[137] Schöbel J., Karg M., Rosenbach D., Krauss G., Greiner A., Schmalz H. (2016). Patchy Wormlike Micelles with Tailored Functionality by Crystallization-Driven Self-Assembly: A Versatile Platform for Mesostructured Hybrid Materials. Macromolecules.

[138] Schöbel J., Hils C., Weckwerth A., Schlenk M., Bojer C., Stuart M.C.A., Breu J., Förster S., Greiner A., Karg M. (2018). Strategies for the selective loading of patchy worm-like micelles with functional nanoparticles. Nanoscale.

[139] Oliver A.M., Gwyther J., Winnik M.A., Manners I. (2017). Cylindrical Micelles with “Patchy” Coronas from the Crystallization-Driven Self-Assembly of ABC Triblock Terpolymers with a Crystallizable Central Polyferrocenyldimethylsilane Segment. Macromolecules.

[140] Oliver A.M., Spontak R.J., Manners I. (2019). Solution self-assembly of ABC triblock terpolymers with a central crystallizable poly(ferrocenyldimethylsilane) core-forming segment. Polym. Chem..

[141] Nunns A., Whittell G.R., Winnik M.A., Manners I. (2014). Crystallization-Driven Solution Self-Assembly of *μ*-ABC Miktoarm Star Terpolymers with Core-Forming Polyferrocenylsilane Blocks. Macromolecules.

[142] Gegenhuber T., Gröschel A.H., Löbling T.I., Drechsler M., Ehlert S., Förster S., Schmalz H. (2015). Noncovalent Grafting of Carbon Nanotubes with Triblock Terpolymers: Toward Patchy 1D Hybrids. Macromolecules.

[143] Finnegan J.R., Lunn D.J., Gould O.E.C., Hudson Z.M., Whittell G.R., Winnik M.A., Manners I. (2014). Gradient Crystallization-Driven Self-Assembly: Cylindrical Micelles with “Patchy” Segmented Coronas via the Coassembly of Linear and Brush Block Copolymers. J. Am. Chem. Soc..

[144] Cruz M., Xu J., Yu Q., Guerin G., Manners I., Winnik M.A. (2018). Visualizing Nanoscale Coronal Segregation in Rod-Like Micelles Formed by Co-Assembly of Binary Block Copolymer Blends. Macromol. Rapid Commun..

[145] Xu J., Zhou H., Yu Q., Manners I., Winnik M.A. (2018). Competitive Self-Assembly Kinetics as a Route To Control the Morphology of Core-Crystalline Cylindrical Micelles. J. Am. Chem. Soc..

[146] Song S., Liu X., Nikbin E., Howe J.Y., Yu Q., Manners I., Winnik M.A. (2021). Uniform 1D Micelles and Patchy & Block Comicelles via Scalable, One-Step Crystallization-Driven Block Copolymer Self-Assembly. J. Am. Chem. Soc..

[147] Bahrami R., Löbling T.I., Gröschel A.H., Schmalz H., Müller A.H.E., Altstädt V. (2014). The Impact of Janus Nanoparticles on the Compatibilization of Immiscible Polymer Blends under Technologically Relevant Conditions. ACS Nano.

[148] Bärwinkel S., Bahrami R., Löbling T.I., Schmalz H., Müller A.H.E., Altstädt V. (2016). Polymer Foams Made of Immiscible Polymer Blends Compatibilized by Janus Particles-Effect of Compatibilization on Foam Morphology. Adv. Eng. Mater..

[149] Yang Q., Loos K. (2017). Janus nanoparticles inside polymeric materials: Interfacial arrangement toward functional hybrid materials. Polym. Chem..

[150] Bahrami R., Löbling T.I., Schmalz H., Müller A.H.E., Altstädt V. (2017). Synergistic effects of Janus particles and triblock terpolymers on toughness of immiscible polymer blends. Polymer.

[151] Walther A., André X., Drechsler M., Abetz V., Müller A.H.E. (2007). Janus Discs. J. Am. Chem. Soc..

[152] Bryson K.C., Löbling T.I., Müller A.H.E., Russell T.P., Hayward R.C. (2015). Using Janus Nanoparticles To Trap Polymer Blend Morphologies during Solvent-Evaporation-Induced Demixing. Macromolecules.

[153] Ruhland T.M., Gröschel A.H., Walther A., Müller A.H.E. (2011). Janus Cylinders at Liquid–Liquid Interfaces. Langmuir.

[154] Walther A., Drechsler M., Müller A.H.E. (2009). Structures of amphiphilic Janus discs in aqueous media. Soft Matter.

[155] Walther A., Matussek K., Müller A.H.E. (2008). Engineering Nanostructured Polymer Blends with Controlled Nanoparticle Location using Janus Particles. ACS Nano.

[156] Walther A., Hoffmann M., Müller A.H.E. (2008). Emulsion Polymerization Using Janus Particles as Stabilizers. Angew. Chem. Int. Ed..

[157] Ruhland T.M., Gröschel A.H., Ballard N., Skelhon T.S., Walther A., Müller A.H.E., Bon S.A.F. (2013). Influence of Janus Particle Shape on Their Interfacial Behavior at Liquid–Liquid Interfaces. Langmuir.

[158] Gröschel A.H., Löbling T.I., Petrov P.D., Müllner M., Kuttner C., Wieberger F., Müller A.H.E. (2013). Janus Micelles as Effective Supracolloidal Dispersants for Carbon Nanotubes. Angew. Chem. Int. Ed..

[159] Schmelz J., Pirner D., Krekhova M., Ruhland T.M., Schmalz H. (2013). Interfacial activity of patchy worm-like micelles. Soft Matter.

[160] Böker A., He J., Emrick T., Russell T.P. (2007). Self-assembly of nanoparticles at interfaces. Soft Matter.

[161] Dai X., Qiang X., Hils C., Schmalz H., Gröschel A.H. (2021). Frustrated Microparticle Morphologies of a Semicrystalline Triblock Terpolymer in 3D Soft Confinement. ACS Nano.

[162] Gegenhuber T., Krekhova M., Schöbel J., Gröschel A.H., Schmalz H. (2016). “Patchy” Carbon Nanotubes as Efficient Compatibilizers for Polymer Blends. ACS Macro Lett..

[163] Ruhland T.M. (2009). Janus Cylinders at Interfaces.

[164] Haruta M. (2003). When Gold Is Not Noble: Catalysis by Nanoparticles. Chem. Rec..

[165] Daniel M.-C., Astruc D. (2004). Gold Nanoparticles: Assembly, Supramolecular Chemistry, Quantum-Size-Related Properties, and Applications toward Biology, Catalysis, and Nanotechnology. Chem. Rev..

[166] Shenhar R., Norsten T.B., Rotello V.M. (2005). Polymer-Mediated Nanoparticle Assembly: Structural Control and Applications. Adv. Mater..

[167] Campelo J.M., Luna D., Luque R., Marinas J.M., Romero A.A. (2009). Sustainable Preparation of Supported Metal Nanoparticles and Their Applications in Catalysis. ChemSusChem.

[168] Rycenga M., Cobley C.M., Zeng J., Li W., Moran C.H., Zhang Q., Qin D., Xia Y. (2011). Controlling the Synthesis and Assembly of Silver Nanostructures for Plasmonic Applications. Chem. Rev..

[169] Giljohann D.A., Seferos D.S., Daniel W.L., Massich M.D., Patel P.C., Mirkin C.A. (2010). Gold Nanoparticles for Biology and Medicine. Angew. Chem. Int. Ed..

[170] Dykman L., Khlebtsov N. (2011). Gold nanoparticles in biomedical applications: Recent advances and perspectives. Chem. Soc. Rev..

[171] Saha K., Agasti S.S., Kim C., Li X., Rotello V.M. (2012). Gold Nanoparticles in Chemical and Biological Sensing. Chem. Rev..

[172] Astruc D., Lu F., Aranzaes J.R. (2005). Nanoparticles as Recyclable Catalysts: The Frontier between Homogeneous and Heterogeneous Catalysis. Angew. Chem. Int. Ed..

[173] Burda C., Chen X., Narayanan R., El-Sayed M.A. (2005). Chemistry and Properties of Nanocrystals of Different Shapes. Chem. Rev..

[174] Murphy C.J., Sau T.K., Gole A.M., Orendorff C.J., Gao J., Gou L., Hunyadi S.E., Li T. (2005). Anisotropic Metal Nanoparticles: Synthesis, Assembly, and Optical Applications. J. Phys. Chem. B.

[175] Lu A.-H., Salabas E.-L., Schüth F. (2007). Magnetic Nanoparticles: Synthesis, Protection, Functionalization, and Application. Angew. Chem. Int. Ed..

[176] Wang J., Li W., Zhu J. (2014). Encapsulation of inorganic nanoparticles into block copolymer micellar aggregates: Strategies and precise localization of nanoparticles. Polymer.

[177] Zhou T., Dong B., Qi H., Mei S., Li C.Y. (2014). Janus hybrid hairy nanoparticles. J. Polym. Sci. Part B Polym. Phys..

[178] Zhang Y., Pearce S., Eloi J.-C., Harniman R.L., Tian J., Cordoba C., Kang Y., Fukui T., Qiu H., Blackburn A. (2021). Dendritic Micelles with Controlled Branching and Sensor Applications. J. Am. Chem. Soc..

[179] Liu L., Corma A. (2018). Metal Catalysts for Heterogeneous Catalysis: From Single Atoms to Nanoclusters and Nanoparticles. Chem. Rev..

[180] Van Deelen T.W., Mejía C.H., de Jong K.P. (2019). Control of metal-support interactions in heterogeneous catalysts to enhance activity and selectivity. Nat. Catal..

[181] Li Z., Ji S., Liu Y., Cao X., Tian S., Chen Y., Niu Z., Li Y. (2020). Well-Defined Materials for Heterogeneous Catalysis: From Nanoparticles to Isolated Single-Atom Sites. Chem. Rev..

[182] Ishida T., Murayama T., Taketoshi A., Haruta M. (2019). Importance of Size and Contact Structure of Gold Nanoparticles for the Genesis of Unique Catalytic Processes. Chem. Rev..

[183] Shifrina Z.B., Matveeva V.G., Bronstein L.M. (2020). Role of Polymer Structures in Catalysis by Transition Metal and Metal Oxide Nanoparticle Composites. Chem. Rev..

[184] Cai J., Li C., Kong N., Lu Y., Lin G., Wang X., Yao Y., Manners I., Qiu H. (2019). Tailored multifunctional micellar brushes via crystallization-driven growth from a surface. Science.

[185] Astruc D. (2020). Introduction: Nanoparticles in Catalysis. Chem. Rev..

[186] Hils C., Dulle M., Sitaru G., Gekle S., Schöbel J., Frank A., Drechsler M., Greiner A., Schmalz H. (2020). Influence of patch size and chemistry on the catalytic activity of patchy hybrid nonwovens. Nanoscale Adv..

[187] Schöbel J., Burgard M., Hils C., Dersch R., Dulle M., Volk K., Karg M., Greiner A., Schmalz H. (2016). Bottom-Up Meets Top-Down: Patchy Hybrid Nonwovens as an Efficient Catalysis Platform. Angew. Chem. Int. Ed..

[188] Mitschang F., Schmalz H., Agarwal S., Greiner A. (2014). Tea-Bag-Like Polymer Nanoreactors Filled with Gold Nanoparticles. Angew. Chem. Int. Ed..

[189] Taguchi T., Isozaki K., Miki K. (2012). Enhanced Catalytic Activity of Self-Assembled-Monolayer-Capped Gold Nanoparticles. Adv. Mater..

[190] Raffa P., Evangelisti C., Vitulli G., Salvadori P. (2008). First examples of gold nanoparticles catalyzed silane alcoholysis and silylative pinacol coupling of carbonyl compounds. Tetrahedron Lett..

[191] Mitsudome T., Yamamoto Y., Noujima A., Mizugaki T., Jitsukawa K., Kaneda K. (2013). Highly Efficient Etherification of Silanes by Using a Gold Nanoparticle Catalyst: Remarkable Effect of O_2_. Chem. Eur. J..

[192] Kronawitt J., Dulle M., Schmalz H., Agarwal S., Greiner A. (2020). Poly(*p*-xylylene) Nanotubes Decorated with Nonagglomerated Gold Nanoparticles for the Alcoholysis of Dimethylphenylsilane. ACS Appl. Nano Mater..

[193] Isozaki K., Taguchi T., Ishibashi K., Shimoaka T., Kurashige W., Negishi Y., Hasegawa T., Nakamura M., Miki K. (2020). Mechanistic Study of Silane Alcoholysis Reactions with Self-Assembled Monolayer-Functionalized Gold Nanoparticle Catalysts. Catalysts.

[194] Hils C., Fuchs E., Eger F., Schöbel J., Schmalz H. (2020). Converting Poly(Methyl Methacrylate) into a Triple-Responsive Polymer. Chem. Eur. J..

